# In vitro toxicological assessment of PhSeZnCl in human liver cells

**DOI:** 10.1007/s43188-022-00148-y

**Published:** 2022-09-08

**Authors:** Raffaella di Vito, Sara Levorato, Cristina Fatigoni, Mattia Acito, Luca Sancineto, Giovanna Traina, Milena Villarini, Claudio Santi, Massimo Moretti

**Affiliations:** 1 Department of Pharmaceutical Sciences (Unit of Public Health), University of Perugia, Via del Giochetto, 06122 Perugia, Italy; 2 Department of Pharmaceutical Sciences (Group of Catalysis Synthesis and Organic Green Chemistry), University of Perugia, Via del Liceo, 06123 Perugia, Italy; 3 Department of Pharmaceutical Sciences (Unit of Physiology), University of Perugia, Via San Costanzo, 06126 Perugia, Italy; 4 Present Address: European Food Safety Authority, Via Carlo Magno 1A, 43126 Parma, Italy

**Keywords:** Phenylselenenylzinc chloride, HepG2, HepaRG, Comet assay, Apoptosis, Cell cycle

## Abstract

Phenylselenenylzinc chloride (PhSeZnCl) is an air-stable selenolate, easily synthesizable through oxidative insertion of elemental zinc into the Se-halogen bond of the commercially available phenylselenyl chloride. PhSeZnCl was shown to possess a marked GPx-like activity both in NMR and in vitro tests, and to effectively react with cellular thiols, and was supposed for a potential use in the chemotherapy of drug-resistant cancers. However, activity of PhSeZnCl in hepatic cells has never been tested before now. In this in vitro approach, we evaluated the cytotoxic, genotoxic, and apoptotic activities, as well as the effects on cell cycle of PhSeZnCl in two preclinical hepatic models, namely HepG2 and HepaRG cells. Results showed that cell viability of HepG2 and HepaRG cells decreased in a dose-dependent manner, with a more marked effect in HepG2 tumour cells. Moreover, treatment with 50 µg/mL PhSeZnCl caused an increase of primary DNA damage (4 h) and a statistically significant increase of HepG2 cells arrested in G_2_/M phase. In addition, it altered mitochondrial membrane potential and induced chromosomal DNA fragmentation (24 h). In HepaRG cells, PhSeZnCl was able to determine a cell cycle-independent induction of apoptosis. Particularly, 50 µg/mL induced mitochondrial membrane depolarization after 24 h and apoptosis after 4 h treatment. Futhermore, all PhSeZnCl concentrations tested determined a significant increase of apoptotic cells after 24 h. Apoptosis was also highlighted by the detection of active Caspase-3 by Western Blot analysis after 24 h exposure. In conclusion, this first toxicological assessment provides new insights into the biological activity of PhSeZnCl in preclinical hepatic models that will be useful in future safety assessment investigation of this compound as a potential pharmaceutical.

**Supplementary Information:**

The online version contains supplementary material available at 10.1007/s43188-022-00148-y.

## Introduction

Selenium (Se)—a trace element discovered in 1817 by Jöns Jakob Berzelius—is both a toxic mineral and an essential micronutrient, with a fairly narrow therapeutic window [1]. Its essentiality to mammals was recognised in the 50’s [2], and in 1973 was reported the identification of the first selenoprotein, glutathione peroxidase (GPx) [3, 4]. At present, at least 25 genes coding for selenoproteins containing the amino acid selenocysteine at their active sites are known in humans [5], with roles, amongst other functions, as antioxidants (GPxs), in maintaining intracellular redox status (thioredoxin reductases), and in thyroid hormone production (iodothyronine deiodinases) [6, 7]. Earlier investigations of Se focused on its inorganic form. Indeed, a cancer chemopreventive activity of Se (i.e., sodium selenide) was proposed in late 60’s [8]. However, although inorganic Se has a good antitumor activity, its marked toxicity, low biocompatibility, and other flaws preclude its straightforward clinical use. Therefore, the interest has moved toward organo-seleno compounds [9]. Capabilities of Se compounds in inhibiting tumour growth and in inducing tumour cell apoptosis have been then widely demonstrated and Se was suggested to have a potential role in cancer chemoprevention [10], with Se chemopreventive activity reported to be completely separable from its nutritional requirements [11]. Furthermore, Se has been shown to possess a unique behaviour exerted by a dual mechanism: (1) via a pro-oxidant pathway, as seen in cytotoxicity and apoptosis of cancer cells, or (2) via an antioxidant pathway, as proposed in cancer chemoprevention in healthy cells [12]. Interestingly, data retrieved in the literature indicate that cancer cells are substantially more sensitive to Se and more prone to apoptosis induction than normal/healthy cells [13, 14].

Among organo-seleno compounds, phenylselenenylzinc chloride (PhSeZnCl) is an air-stable selenolate, easily synthesizable through oxidative insertion of elemental zinc into the Se-halogen bond of the commercially available phenylselenyl chloride (PhSeCl) [15, 16]. PhSeZnCl was shown to possess a marked GPx-like activity both in NMR and in vitro tests, and demonstrated to effectively react with cellular thiols [17–19], and was supposed for a possible use as a cytotoxic agent in the chemotherapy of drug-resistant cancers [17].

However, to the best of our knowledge, activity of PhSeZnCl in hepatic cells has never been tested before now. As most of market withdrawal of pharmaceutical products is related to hepatotoxicity [20], it is essential to carry out in vitro hepatic toxicity assessment since early phases of investigation. In this study, we have evaluated the cytotoxic, genotoxic, and apoptotic activities, as well as effects on cell cycle of PhSeZnCl in two preclinical hepatic models, namely HepG2 and HepaRG cells [21].

## Materials and methods

### Chemicals, reagents and media

All reagents used were of analytical grade. Ethanol, ethylenediaminetetracetic acid disodium (Na_2_EDTA) and tetrasodium (Na_4_EDTA) salt, sodium chloride (NaCl), and sodium hydroxide (NaOH) were purchased from Carlo Erba Reagenti Srl (Milan, Italy). Dimethyl sulfoxide (DMSO), ethidium bromide, hydrocortisone hemisuccinate, insulin, low- and normal melting-point agarose (LMPA and NMPA, respectively), 4-nitroquinoline *N*-oxide (4NQO), staurosporine, tris(hydroxymethyl)aminomethane (Tris base), Triton X-100, trypan blue, and valinomycin were obtained from Sigma-Aldrich Srl (Milan, Italy). Acridine orange (AO), 6,4′-diamidino-2-phenylindole (DAPI), 5,5′,6,6′-tetrachloro-1,1′,3,3′-tetraethylbenzimidazolecarbocyanine iodide (JC-1), Via1-Cassette™, and NC-Slide A8™ were purchased from ChemoMetec A/S (Allerød, Denmark). Eagle’s Minimum Essential Medium (MEM), and Dulbecco’s phosphate-buffered saline, pH 7.4 (PBS) were purchased from Invitrogen Srl (Milan, Italy). Countess™ cell counting chamber slides, Enhanced chemiluminesce (ECL) detection kit, Gibco™ William’s E medium, Glutamax and Pierce™ IP lysis buffer were from Thermo Fisher Scientific (Waltham, MA, USA). Antibiotics (penicillin and streptomycin), foetal bovine serum (FBS), L-glutamine, MEM non-essential amino acids (NEAA), sodium pyruvate, and trypsin were purchased from Euroclone SpA (Milan, Italy). Anti-Caspase-3 (E-AB-60017) and anti-β-Actin (E-AB-20031) antibodies were purchased from Elabscience (Houston, TX, USA). Anti-mouse and anti-rabbit horseradish peroxidase (HRP)-linked secondary antibodies were purchased from Cell Signaling Technology (Danvers, MA, USA). Conventional microscope slides and coverslips were supplied by Knittel-Glaser GmbH (Braunschweig, Germany). Distilled water was used throughout the experiments.

### Phenylselenenylzinc chloride (PhSeZnCl)

**Fig. 1:**
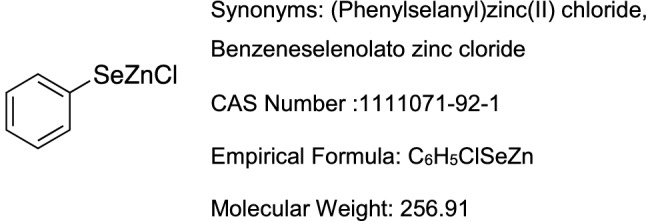
PhSeZnCl chemical data

PhSeZnCl (Fig. 1) was synthesised in the laboratory of Professor Claudio Santi as previously described [16] by refluxing equimolar amounts of PhSeCl and freshly activated zinc in dry THF. The top concentration used was the highest soluble concentration obtained—as previously described [22]—according to the US National Toxicology Program Interagency Center for the Evaluation of Alternative Toxicological Methods (NICEATM) [23] and abiding by the US Food and Drug Administration [24] and OECD guidelines for testing pharmaceuticals intended for human use [25].

Briefly, PhSeZnCl dry powder was firstly suspended in DMSO until the concentration of 50 mg/mL, and then further diluted in complete medium until desired concentrations, namely 500 µg/mL.

### Cell lines and culture conditions

This research aimed to evaluate the cytotoxic, genotoxic, and apoptotic activities, as well as effects on cell cycle of PhSeZnCl in two preclinical hepatic models, namely HepG2 and HepaRG cells.

HepG2 cells originate from a clone of human tumour cells isolated in 1975 from the liver of a young Argentine of 15 years with a diagnosis of hepatoblastoma [26–28]. These cells are commonly used for toxicity investigation due to their unlimited life span, stable phenotype, high availability, and easy handling [29]. Moreover, it has been shown that the use of such an in vitro model is more adherent to the situation in vivo, compared with the addition of exogenous metabolic activation systems (e.g., S9-mix) in the case of use of other lines with lower metabolic capabilities [30].

The human HepG2 cell line (ATCC HB 8065) was obtained from Istituto Zooprofilattico Sperimentale della Lombardia e dell’Emilia Romagna “Bruno Ubertini” (Brescia, Italy). The cells were grown as monolayer cultures in 25 cm^2^ tissue flasks in MEM supplemented with 10% (v/v) FBS, 1% NEAA, 1 mM sodium pyruvate, 100 U/mL penicillin and 0.1 mg/mL streptomycin, at 37 °C in a humidified atmosphere containing 5% CO_2_. HepG2 cells were sub-cultured by dispersal with 0.05% trypsin in 0.02% Na_4_EDTA for a contact time of 5 min and replated at a 1:2 dilution to maintain the cells in the exponential growth phase. For cell treatment, sub-confluent HepG2 cultures were collected by trypsin treatment and suspended in complete MEM culture medium.

The experimental design also included tests on undifferentiated HepaRG™ liver cells. HepaRG cells derive from a tumour of a female patient suffering from chronic hepatitis C infection and hepatocellular carcinoma [31]. Regardless of their differentiation status, HepaRG cells are closer to primary human hepatocytes and liver tissues compared with HepG2 cells [32]. For these reasons, HepaRG cells are considered to be a useful model for in vitro studies on drug metabolism and toxicity [33, 34].

The human HepaRG hepatic cells were purchased by Thermo Fisher Scientific (Waltham, MA, USA). The cells were thawed, seeded at low density (2 × 10^4^ cells/cm^2^) in 75 cm^2^ flasks and maintained in William's E medium supplemented with 1% Glutamax, 5 μg/mL human insulin, 50 μM hydrocortisone hemisuccinate, 100 U/mL penicillin, 0.1 mg/mL streptomycin and 10% FBS at 37 °C in a humidified atmosphere containing 5% CO_2_. When high confluence was achieved (at day 14), cells were sub-cultured, in order to maintain the cell line. Medium was renewed every 2 or 3 days.

### Cytotoxicity testing: trypan blue dye exclusion assay

We assessed the possible cytotoxicity of PhSeZnCl by evaluating 6 scalar concentrations (i.e., 6.25, 12.5, 25, 50, 100, and 500 µg/mL); the top concentration was based on the US Food and Drug Administration guidelines for testing pharmaceuticals intended for human use [24]. The trypan blue dye exclusion assay is based on the principle that live cells possess intact cell membranes that exclude certain dyes, such as trypan blue, whereas dead cells do not. The reactivity of trypan blue is based on the fact that the chromophore is negatively charged and does not interact with the cell unless the membrane is damaged; therefore, the cells that exclude the dye are considered viable [35].

For the test, cells (5 × 10^5^ per well) were dispensed within six-well culture plates (Becton Dickinson Italia SpA, Milan, Italy) in 5 mL volumes. Cells were maintained in culture for 24 h to form a semiconfluent monolayer and then treated with 2% Triton X-100 (positive control) or PhSeZnCl over a range of six concentrations. The cells were exposed for 4 or 24 h. Cytotoxicity was measured using a Countess™ (Invitrogen Srl, Milan, Italy) automated cell counter [36]. Briefly, aliquots of cell suspensions were mixed with equal volumes of 0.4% trypan blue and 10 µL loaded onto a Countess cell counting chamber slide. The Countess counter is equipped with a camera that acquires images from cell samples on the chamber slide, the image analysis software automatically analyses the acquired cell images and measures cell count and viability.

### Cytotoxicity testing: AO/DAPI double staining

The number of total and viable cells was also estimated by staining cell populations with AO and DAPI fluorophores. For the test, the cells were cultured as above described for the trypan blue exclusion assay. After cell treatment, aliquots of cell suspensions were loaded into Via1-Cassette. The inside of the Via1-Cassette is coated with AO (staining the entire population of cells) and DAPI (staining nonviable cells). The Via1-Cassettes were then placed in a NucleoCounter^®^ NC-3000™ (Chemometec, Allerød, Denmark), a fluorescence-based image cytometer, where cell concentration and viability were determined [36, 37]. Total cell concentration in Via1-Cassette was displayed on a personal computer using the NucleoView software.

### Genotoxicity testing: comet assay

To avoid conditions that would lead to false-positive results arising from DNA damage associated with cytotoxicity [38], the three highest non-cytotoxic concentrations of PhSeZnCl (i.e., 12.5, 25, and 50 µg/mL) were processed in the comet assay [39]. The test was conducted under alkaline conditions (alkaline unwinding/alkaline electrophoresis, pH > 13) following the original three-layer procedure [39] as described in detail elsewhere [36].

Briefly, 48 h prior to PhSeZnCl treatment, HepG2 and HepaRG subcultures were trypsinized and seeded (approximately 5 × 10^5^ cells/well) in six-well plates. Cells were then treated for 4 h with PhSeZnCl; cell subcultures were also treated with the model mutagen 4NQO (1 µM; positive control). One hundred cells were randomly selected and analysed from each experimental point. The percentage of DNA in the comet tail (i.e., tail intensity %) was used as a measure of the extent of DNA damage [40]. The percentage of hedgehog cells (cells with tail intensity of around 85–90% and above) were reported and hedgehog comets were included in the analysis of genotoxicity [41].

### Analysis of cell cycle

For cell cycle analysis, HepG2 and HepaRG cells were cultured overnight in six-well plates at 5 × 10^5^ cells per well. Cell cultures were then exposed to PhSeZnCl (i.e., 12.5, 25, and 50 µg/mL) for 24 h. At the end of the treatment, the cells were harvested, fixed with 70% ethanol, and maintained at 0–4 °C for at least 24 h. After centrifugation, cell pellets were washed with PBS and treated for 5 min at 37 °C with 0.5 mL of PBS containing 1 µg/mL DAPI and 0.1% Triton X-100. After staining, DAPI fluorescence was quantified by fluorescence microscopy with the automated cytometer NucleoCounter NC-3000 [42]. The stained cells were analysed using the NucleoView NC-3000 software and obtained results were presented in the DNA content histograms where different phases of the cell cycle were demarcated.

### Early apoptosis: evaluation of mitochondrial membrane potential (ΔΨm) assay

The cells were cultured as above and exposed to PhSeZnCl for 4 and 24 h. Valinomycin 0.5 µM was used as a positive control. Following treatment, mitochondrial membrane potential (ΔΨm) was estimated by a NucleoCounter NC-3000 automated system after staining of cells with JC-1 and DAPI [43]. In early apoptotic cells, where the mitochondrial membrane potential collapses, the monomeric JC-1 remains cytosolic and stains the cytosol with a green colour. On the other hand, in non-apoptotic cells, JC-1 rapidly forms complexes with intense red fluorescence [36, 44]. Mitochondrial membrane depolarization was revealed as a decrease in the red/green fluorescence intensity ratio. The scatterplots obtained by the NucleoView NC-3000 software were used to demarcate the percentage of polarized/healthy cells, depolarized/early apoptotic cells and DAPI positive/late apoptotic or necrotic cells, respectively [45].

### Late apoptosis: chromosomal DNA fragmentation assay

HepG2 and HepaRG cells were cultured as above described for cell cycle analysis and exposed to PhSeZnCl for 4 and 24 h. Staurosporine (1 μM) was used as a positive control. Fragmentation of chromosomal DNA during late apoptosis was revealed by discrete sub-G_1_ peaks on DNA content histograms [46]. At the end of the treatment, a NucleoCounter NC-3000 automated system using fluorescence microscopy and image analysis was used to determine the extent of DNA fragmentation [45]. For the test, the cells were first permeabilized with ethanol; during this procedure, the low molecular weight DNA inside apoptotic cells leaks out and is removed from the sample during the subsequent washing step. The high molecular weight DNA retained in the cells was stained with DAPI.

### Western immunoblot analysis

Activation of Caspase-3—an effector in most of apoptotic pathways—was also investigated by Western Blot analysis. HepG2 and HepaRG cells were seeded in T25 flasks at 2 × 10^6^ cells/flask and cultured overnight. Cells were exposed to PhSeZnCl (i.e., 12.5, 25, and 50 µg/mL) for 4 and 24 h. Staurosporine (1 μg/mL) was used as positive control. After treatment, cells were scraped and lysed with ice-cold Pierce IP lysis buffer. Lysates were centrifuged at 12,000×*g* and supernatant containing cell proteins was collected. The protein amount in each lysate was determined by Bradford’s method. Sixty micrograms of total proteins for each sample were resolved on a 16% SDS-Page gel and blotted on a nitrocellulose membrane (0.2 μm pore). Membranes were blocked for 1 h at room temperature with 5% skim milk in PBS containing 0.05% Tween-20. For immunodetection, nitrocellulose membranes were incubated overnight with 1:1000 anti-Caspase-3 antibody at 4 °C. β-Actin was used as loading control. Then, membranes were washed in T-PBS and incubated in anti-mouse (for β-Actin) or anti-rabbit (for Caspase-3) (HRP)-linked secondary antibody for 2 h at room temperature. Protein signals were detected using ECL detection kit (Thermo Fisher Scientific) and the ImageQuant LAS 500 (GE Healthcare, Milan, Italy). Densitometric analysis was carried out using ImageJ software (https://imagej.nih.gov/ij/).

### Statistical analysis

The assays were carried out at least in triplicate. After testing the normal distribution of data with the Kolmogorov–Smirnov test, the results were expressed as the mean ± standard error of the mean (SEM). Differences were investigated by one-way analysis of variance (ANOVA) followed by Dunnett’s post hoc analysis for pairwise comparisons between PhSeZnCl and untreated (control) cells. Student’s *t*-test was used for positive controls. The level of significance was set at *p* < 0.05. IC_50_ was estimated by considering logarithm transformed PhSeZnCl concentrations.

## Results

### Cytotoxicity

**Fig. 2:**
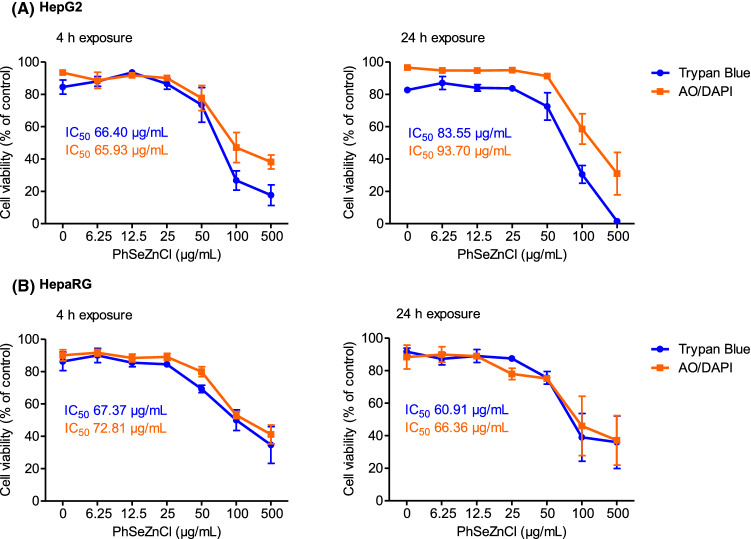
Effects of PhSeZnCl on cell viability in HepG2 [*A*] and HepaRG [*B*] cells after 4- or 24-h exposure. Concentration and time-dependent effects of PhSeZnCl on cell viability were assessed by the Trypan blue dye exclusion assay and AO/DAPI double staining. Results are summarized as mean ± SEM. IC_50_ was estimated by considering logarithm transformed PhSeZnCl concentrations

For cytotoxicity testing, the cells were exposed for 4 and 24 h to PhSeZnCl over a range of 6 scalar concentrations, from 6.25 to 500 µg/mL. Viability of cells was evaluated by trypan blue dye exclusion assay and double staining with AO/DAPI fluorochromes. The results showed that the cell viability of HepG2 and HepaRG cells decreased in a dose-dependent manner (Fig. 2), with a more marked cytotoxic effect of PhSeZnCl in HepG2 tumour cells. Microphotographs and plots are reported in Supplementary Figs. 1A/B, 2A/B, 3A/B and 4A/B.

The results of cytotoxicity assays determined the choice of concentrations to be evaluated afterwards. The next steps were then conducted using the three highest concentrations, which did not show cytotoxic effects in Trypan blue and AO/DAPI assays (i.e., 12.5, 25, and 50 µg/mL). Particularly, we considered as cytotoxic those concentrations that led to cell viability lower than 55 ± 5%, in accordance with OECD guidelines [25].

### Genotoxicity

Table 1 summarizes the results of the comet assay. Exposure of HepG2 cells to PhSeZnCl significantly increased the extent of DNA damage only at the highest tested concentration (i.e., 50 µg/mL; *p* = 0.024), compared with untreated cells (negative control). Furthermore, the 50 µg/mL tested dose also showed a statistically significant increased frequency of hedgehog cells (*p* < 0.001). Whereas exposure of HepaRG cells to PhSeZnCl did not induce any increase in the extent of primary DNA damage, nor in the frequency of hedgehog cells. Representative microphotographs are illustrated in Supplementary Figs. 5–6.

**Table 1:** Primary DNA damage in HepG2 and HepaRG cells exposed for 4 h to different concentrations of PhSeZnCl

PhSeZnCl (µg/mL)	HepG2		HepaRG
	Tail intensity (% DNA)	Hedgehog cells (%)	Tail intensity (% DNA)
0	1.38 ± 0.04	3.43 ± 1.13	0.69 ± 0.19
12.5	1.66 ± 0.27	2.43 ± 0.61	0.57 ± 0.22
25	2.07 ± 0.11	3.29 ± 0.99	0.55 ± 0.02
50	5.55 ± 2.02*	31.00 ± 7.60*	0.83 ± 0,12
Positive control^a^	7.54 ± 1.00*	5.71 ± 2.07	9.90 ± 3.69*

Extent of DNA strand breakage is expressed in terms of tail intensity (% DNA migrated in the comet tail); % number of hedgehog cells is also reported. Results of each experimental set are summarized as the mean value of at least five independent experiments (± SEM)

Statistical analysis: * indicates significantly higher levels of primary DNA damage or number of hedgehog cells compared with the negative control (*p* < 0.05), one-way ANOVA followed by Dunnett’s post hoc analysis. Student’s *t*-test was used for positive controls

^a^4NQO 1 µM

### Cell cycle analysis

**Fig. 3:**
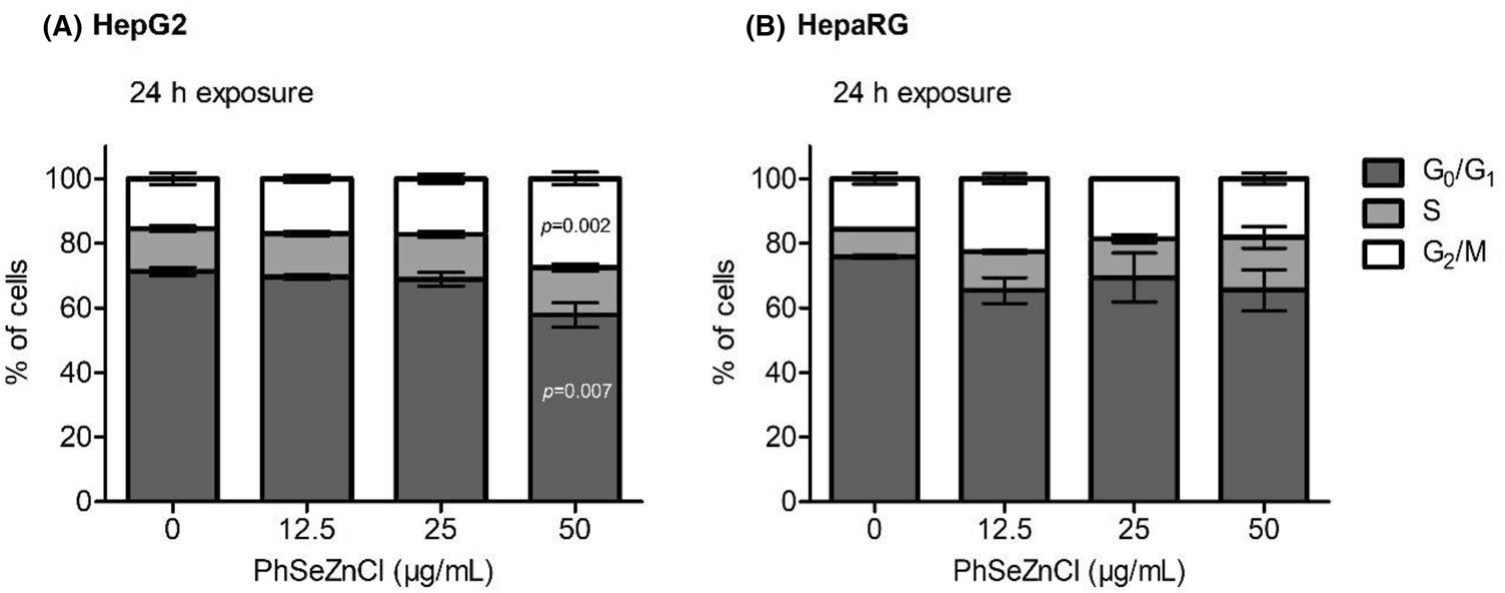
Effects of PhSeZnCl on HepG2 [*A*] and HepaRG [*B*] cell cycle determined using the three highest concentrations which did not show cytotoxic effects. Statistical analysis: *p* values indicate statistically significant different proportion of cells in the cell cycle phases, compared with the negative control, one-way ANOVA followed by Dunnett’s post hoc analysis

The inhibition of cell proliferation is significantly characterized by interference with the normal cell cycle. The distribution of treated and control cells in different phases of the cell cycle is presented in Fig. 3 and in Supplementary Figs. 7–8. Following treatment with 50 µg/mL PhSeZnCl, the percentage of cells in the G_2_/M phase significantly increased to 26.7%, compared with untreated cells (15.1%; *p* = 0.002); concurrently, the increase in G_2_/M population was accompanied by a statistically significant decrease of cells in the G_0_/G_1_ phase (56.0%), compared with untreated control (69.3%; *p* = 0.007). No statistically significant differences were observed in HepaRG cells exposed to PhSeZnCl.

### Apoptosis

Apoptosis was determined using the three highest concentrations which did not show cytotoxic effects. Loss of mitochondrial membrane potential (ΔΨm) and the induction of DNA fragmentation were investigated using the fluorescence image cytometer NucleoCounter NC-3000. Early and late apoptosis results are expressed as percentage of apoptotic cells and are summarized in Tables 2 and 3, respectively; valinomycin (0.5 µM) and staurosporine (1 µM) were used as a positive control in the mitochondrial membrane potential (ΔΨm) assay and in the chromosomal DNA fragmentation assay, respectively. At its highest tested concentration (i.e., 50 µg/mL), PhSeZnCl induced a statistically significant increase of HepG2 cells in early and late apoptosis after 24 h of treatment, as compared with control cells.

**Table 2:** Effects of PhSeZnCl on early (mitochondrial membrane depolarization; ΔΨm) apoptosis induction in HepG2 and HepaRG cells; experimental groups comprised cells treated for 4 and 24 h

PhSeZnCl (µg/mL)	HepG2		HepaRG	
	4 h	24 h	4 h	24 h
12.5	− 1.16 ± 1.01	− 5.14 ± 1.88	− 3.88 ± 5.95	0.84 ± 1.05
25	1.62 ± 0.74	− 4.48 ± 0.75	16.78 ± 14.04	7.44 ± 7.23
50	2.11 ± 1.17	8.55 ± 1.12*	22.30 ± 9.82	18.99 ± 8.04*
Positive control^a^	23.09 ± 5.11*	14.95 ± 3.63*	56.18 ± 6.31*	34.09 ± 13.37*

Results are expressed as increase of apoptotic cells (%) with respect to negative control and are summarized as the mean value of four independent experiments (± SEM)

Statistical analysis: * indicates significantly higher levels of apoptosis compared with the negative control (*p* < 0.05), one-way ANOVA followed by Dunnett’s post hoc analysis. Student’s *t*-test was used for positive controls

^a^Valinomycin 0.5 µM

**Table 3:** Effects of PhSeZnCl on late (chromosomal DNA fragmentation) apoptosis induction in HepG2 and HepaRG cells; experimental groups comprised cells treated for 4, and 24 h

PhSeZnCl (µg/mL)	HepG2		HepaRG	
	4 h	24 h	4 h	24 h
12.5	0.55 ± 3.14	− 1.62 ± 1.73	14.04 ± 7.18	14.44 ± 5.77*
25	2.01 ± 3.19	− 0.25 ± 1.29	19.48 ± 14.72	27.60 ± 4.35*
50	5.43 ± 2.37	10.33 ± 1.58*	35.49 ± 9.53*	35.81 ± 4.98*
Positive control^a^	21.88 ± 5.40*	24.24 ± 6.99*	32.27 ± 10.70*	47.46 ± 8.09*

Results are expressed as increase of apoptotic cells (%) with respect to negative control and are summarized as the mean value of four independent experiments (± SEM)

Statistical analysis: * indicates significantly higher levels of apoptosis compared with the negative control (*p* < 0.05), one-way ANOVA followed by Dunnett’s post hoc analysis. Student’s *t*-test was used for positive controls

^a^Staurosporine 1 µM

When considering HepaRG cells, after 24 h treatment mitochondrial membrane depolarization was induced by the highest concentration. Apoptosis induction was also confirmed by DNA fragmentation assay. Indeed, after 24 h treatment with all PhSeZnCl concentrations tested (12.5, 25 and 50 µg/mL), significant increase of apoptotic cells was observed. The highest concentration was also able to trigger DNA fragmentation after 4 h, as well. Plots are extensively illustrated in Supplementary Figs. 9A/B, 10A/B, 11A/B and 12A/B.

### Western Blot

**Fig. 4:**
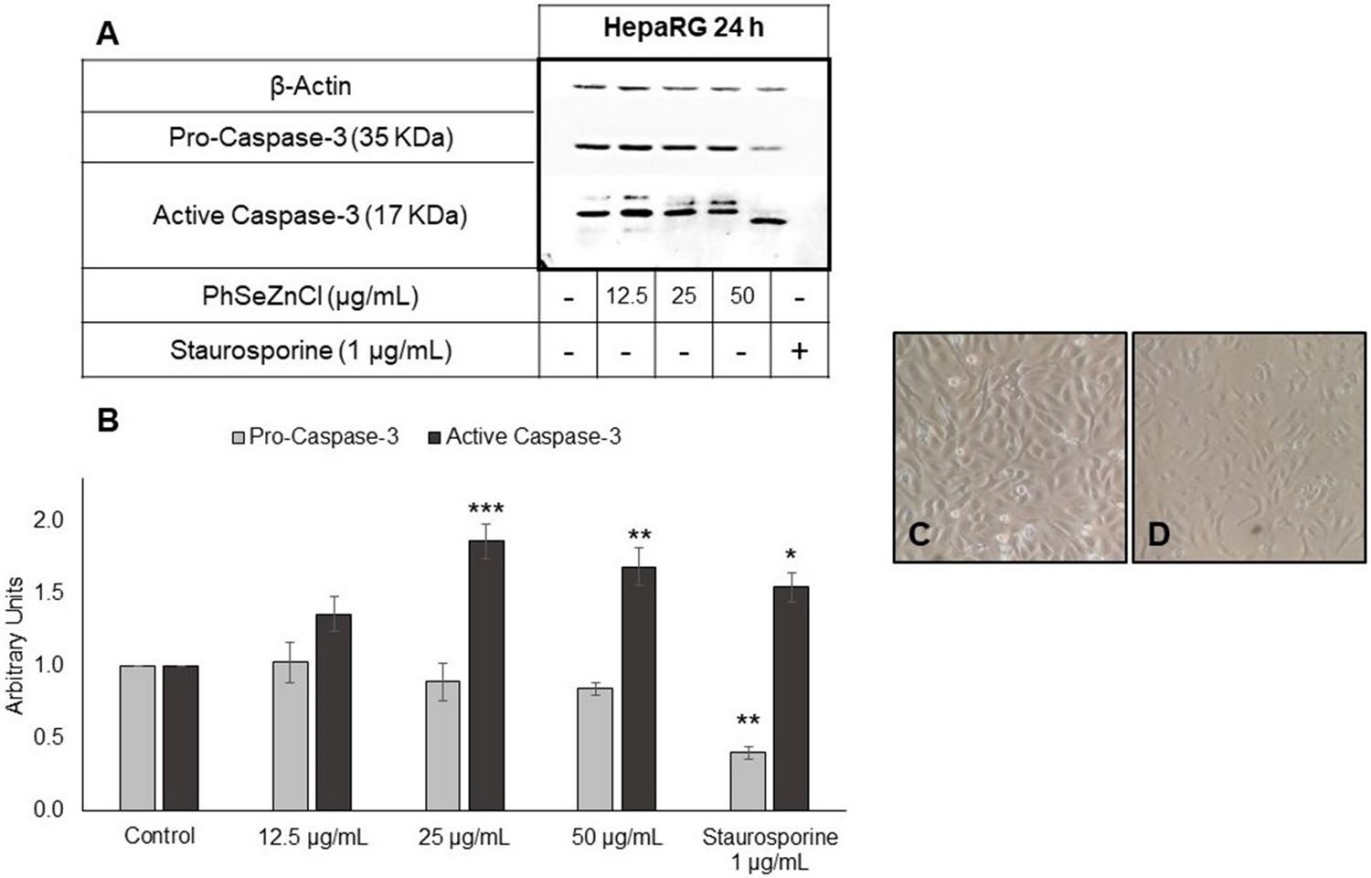
Western Blot analysis for the detection of pro- and activated Caspase-3. [**A**] 24 h treated HepaRG cells: representative image; [**B**] results (normalized to β-Actin) are presented as arbitrary units relative to control, taken the latter as a unit. Statistical analysis: one-way ANOVA followed by Dunnett’s post hoc analysis; Student’s *t*-test was used for positive controls; *p ≤ 0.05, **p ≤ 0.01, ***p ≤ 0.001. [**C**, **D**] microphotographs of HepaRG cells: untreated (control) and 50 µg/mL treated cells, respectively

Western Blot results and photographs are reported in Fig. 4. For HepG2 cells (both at 4 and 24 h) and HepaRG cells exposed for 4 h to PhSeZnCl we were not able to detect a clear and quantifiable signal for active Caspase-3 (data not shown). Conversely, for HepaRG cells a marked activation of Caspase-3 was observed after 24 h exposure to the two highest tested concentrations.

## Discussion

In this study, we have investigated PhSeZnCl biological activity in two human liver cell lines, represented in the present in vitro approach by HepG2 and HepaRG cells.

In HepG2 cells, PhSeZnCl induced concentration-dependent decrement in cell viability—particularly marked at medium/high concentrations—with an IC_50_ ranging from ca. 65 to 90 µg/mL (~ 250–350 µM). These results are in agreement with previously published data indicating an IC_50_ > 100 µM for PhSeZnCl in LNCap (prostate adenocarcinoma) and A549 (lung carcinoma) tumour cells [17]. When considering HepaRG cells, results showed a concentration-dependent effect, as well, but with a more gradual decline in cell viability. These trends in these two hepatic models are almost superimposable with those reported by Guillouzo and co-workers [47], who challenged HepG2 and undifferentiated HepaRG with diclofenac.

Moreover, PhSeZnCl (50 µg/mL) strongly induced DNA damage in HepG2 liver cells and significantly increased the percentage of hedgehog cells. Conversely, genotoxicity was not observed in HepaRG cells. Depending on the characteristics of the investigated compounds, these two hepatic cell lines have been reported to show different sensitivity—in terms of DNA damage and response to apoptotic stimuli—which might be related to differences in oxidative metabolism, antioxidant and detoxifying capacities [47–49]. This phenomenon highlights the importance of considering more than one in vitro model in the preliminary toxicological assessment of new compounds.

Furthermore, we have analysed the cell cycle profiles, as well as the induction of early and late apoptosis in HepG2 and HepaRG cells after treatment with PhSeZnCl. In HepG2, exposure to PhSeZnCl (24 h, 50 µg/mL) caused a statistically significant increase of cells arrested in G_2_/M phase (the G_2_/M checkpoint serves to prevent the cell from entering mitosis with genomic DNA damage). Moreover, 50 µg/mL PhSeZnCl altered mitochondrial membrane potential (ΔΨm) and induced chromosomal DNA fragmentation after a 24 h treatment.

In HepaRG cells, PhSeZnCl exposure did not interfere with cell cycle distribution. However, it was able to determine cell cycle-independent induction of apoptosis. In particular, the highest concentration induced mitochondrial membrane depolarization after 24 h and DNA fragmentation after 4 h treatment. Moreover, all PhSeZnCl concentrations tested (12.5, 25 and 50 µg/mL), determined a significant increase of apoptotic cells with fragmented DNA. This behaviour is corroborated by Western Blot analysis, which highlighted the presence of activated Caspase-3—an effector in most of apoptotic pathways—after exposure to the two highest tested concentrations. Furthermore, our results suggest that in HepG2 cells the induction of apoptosis occurs more slowly than in HepaRG cells. These results are supported by many studies on organo-seleno compounds which have demonstrated the targeting effects of these molecules on the structure and functions of mitochondria (e.g., by altering the membrane redox potential), which are key regulatory organelles in apoptosis signal transduction pathways [50–52].

In the light of the results observed, this first toxicological assessment revealed a remarkable activity of this seleno-compound towards HepG2 and HepaRG cells.

The ability of PhSeZnCl to inhibit cell proliferation, affect DNA integrity, perturb cell cycle and induce apoptosis in human high-proliferative liver cell lines represents an attractive feature that definitely deserves deeper investigations in a pharmacological context. We hopefully claim that this study would pave the way for further investigations in other in vitro models for liver toxicological assessment, such as primary hepatocytes, three-dimentional culture systems, bioartificial livers and/or co-culture systems.

Previous studies of PhSeZnCl mainly focused on its GPx-like activity to underline its potential anti-cancer effects in different cell lines. However, effects of this seleno-compound in hepatic cells have never been investigated before now.

In summary, this work demonstrated that PhSeZnCl inhibited the proliferation of both HepG2 and HepaRG cells and induced apoptosis at its highest tested concentration. Moreover, it was able to induce genotoxicity and affect cell cycle distribution in HepG2 cell line.

In conclusion, the present study provides new insights into the biological activity of PhSeZnCl in preclinical hepatic models that will be useful in future safety assessment investigation of this compound as a potential pharmaceutical.

## Supplementary Information

Below is the link to the electronic supplementary material.

Supplementary file 1 (PDF 1220 kb)

## Data Availability

The experimental data used to support the findings of this study are included within the article.
