# Supplementary material for: In vitro toxicological assessment of PhSeZnCl in human liver cells
Source: Toxicol Res. 2022 Sep 8;39(1):105–14. doi: 10.1007/s43188-022-00148-y (PMC9839901; doi:10.1007/s43188-022-00148-y)
Supplement: Supplementary file 1 — (PDF 1220 kb) [file 43188_2022_148_MOESM1_ESM.pdf]

[a] Negative control

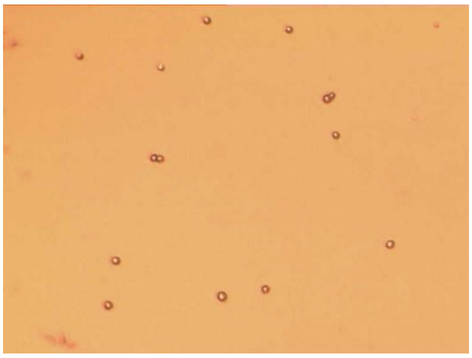

[b] 6.25 µg/mL

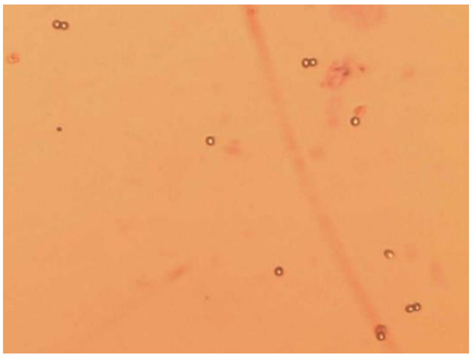

[c] 12.5 µg/mL

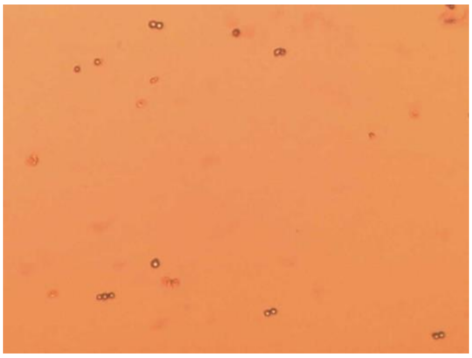

[d] 25 µg/mL

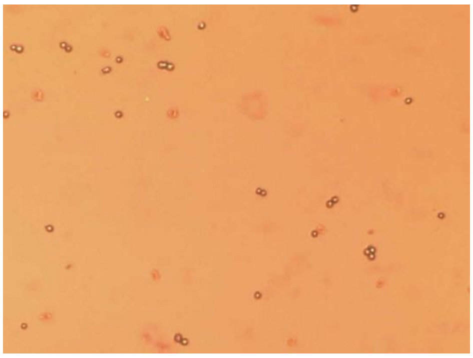

[e] 50 µg/mL

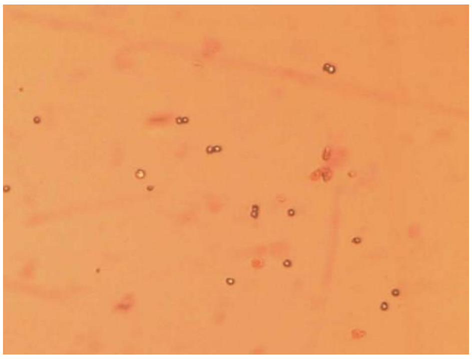

[f] 100 µg/mL

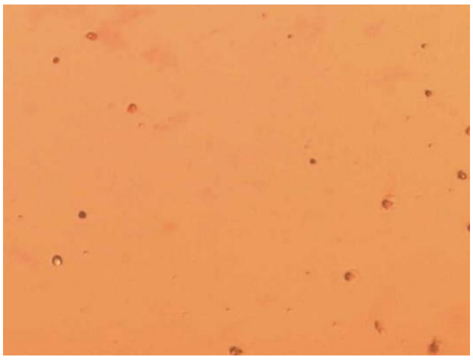

[g] 500 µg/mL

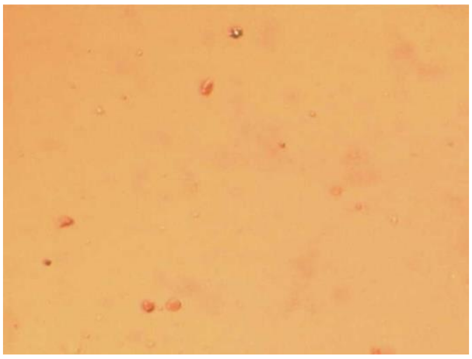

Supplementary Fig. 1/A Effects of PhSeZnCl on cell viability measured by Trypan Blue assay in HepG2 cells after a 4-hour exposure.

[a] Negative control

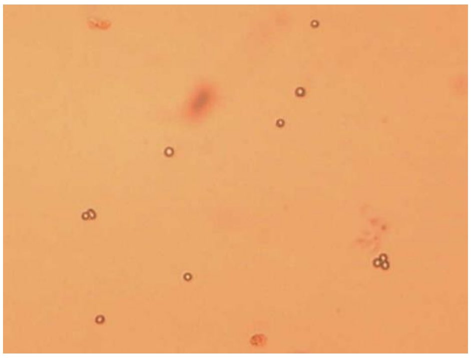

[b] 6.25 µg/mL

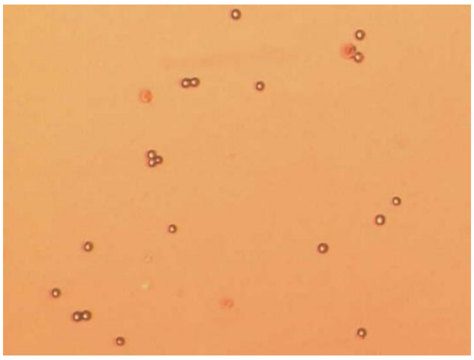

[c] 12.5 µg/mL

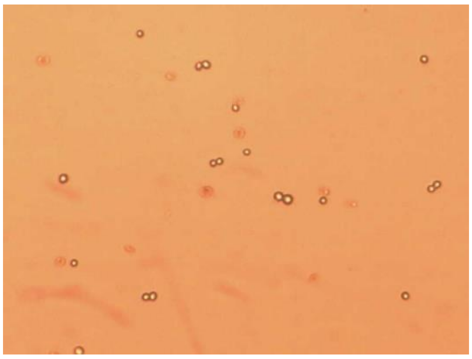

[d] 25 µg/mL

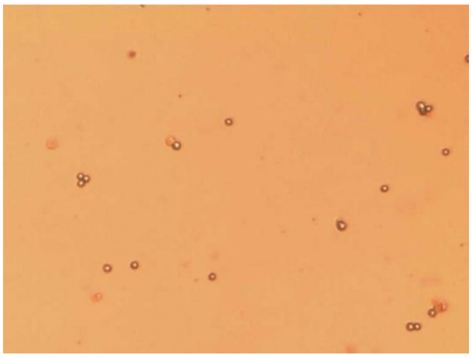

[e] 50 µg/mL

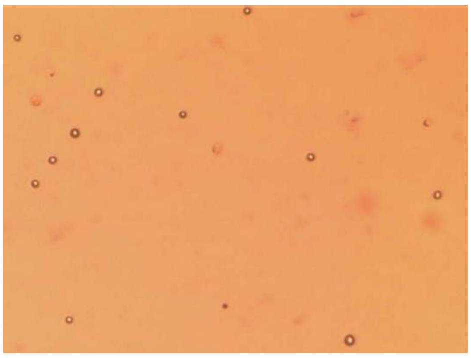

[f] 100 µg/mL

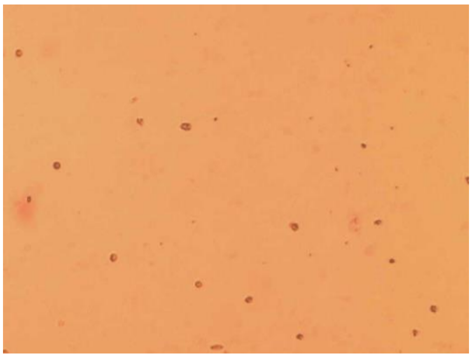

[g] 500 µg/mL

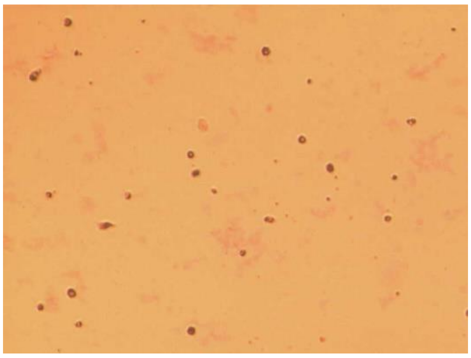

Supplementary Fig. 1/B Effects of PhSeZnCl on cell viability measured by Trypan Blue assay in HepG2 cells after a 24-hour exposure.

[a] Negative control

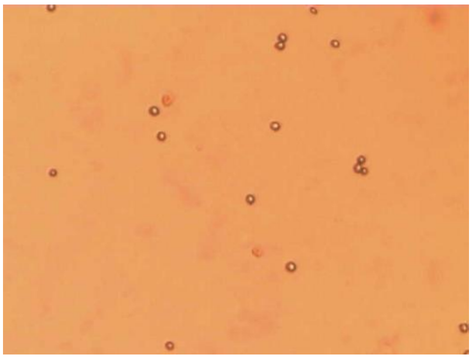

[b] 6.25 μg/mL

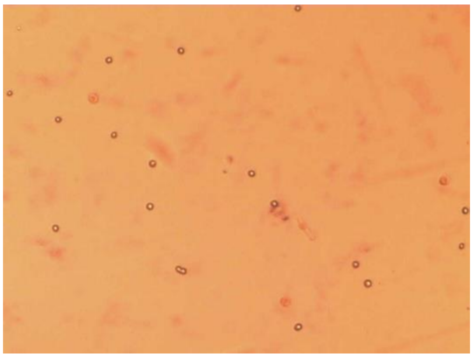

[c] 12.5

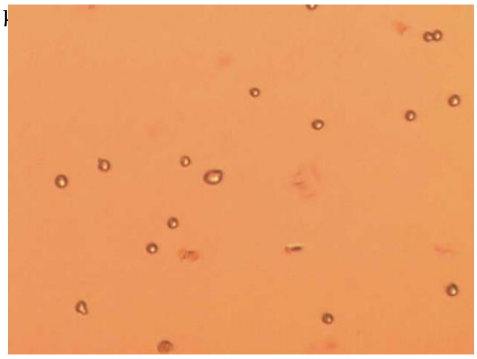

[d] 25 μg/mL

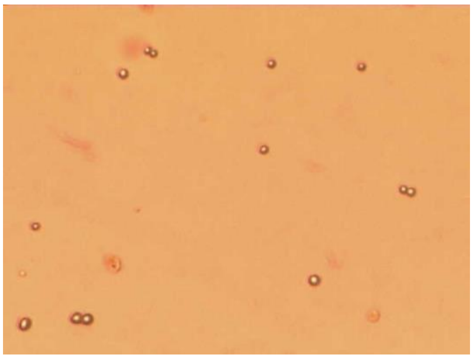

[e] 50 μg/mL

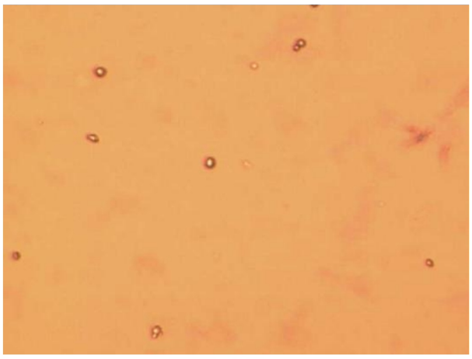

[f] 100 μg/mL

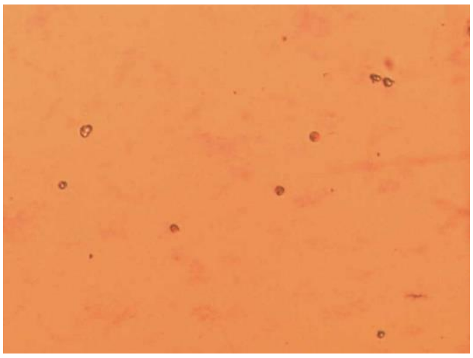

[g] 500 μg/mL

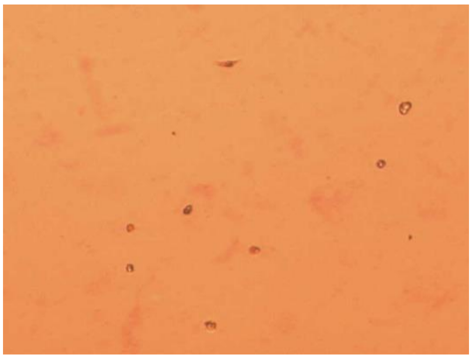

Supplementary Fig. 2/A Effects of PhSeZnCl on cell viability measured by Trypan Blue assay in HepaRG cells after a 4-hour exposure.

[a] Negative control

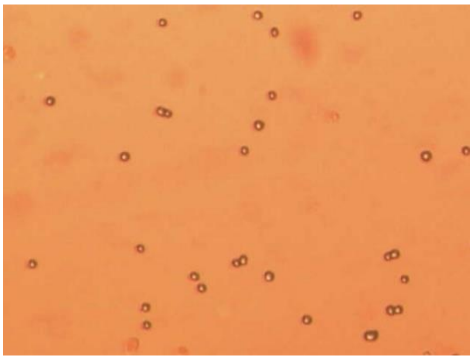

[b] 6.25 µg/mL

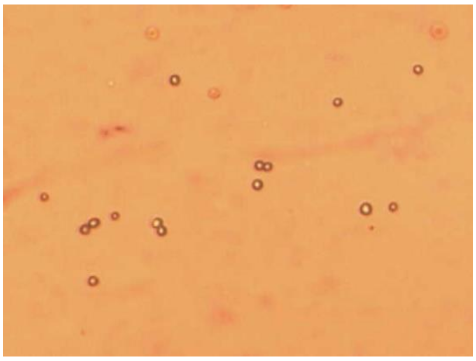

[c] 12.5 µg/mL

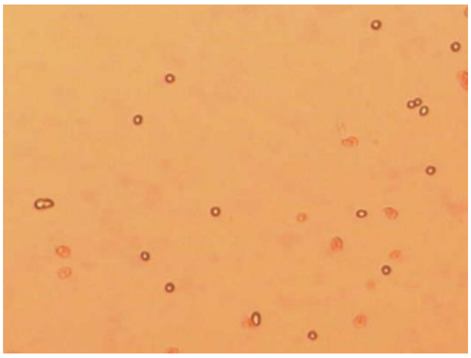

[d] 25 µg/mL

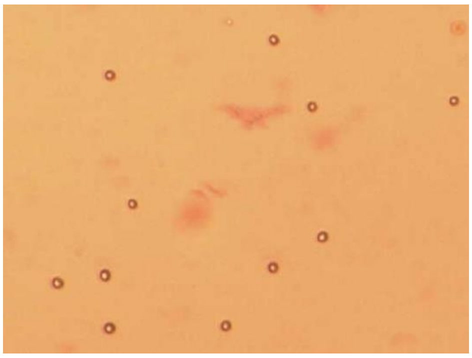

[e] 50 µg/mL

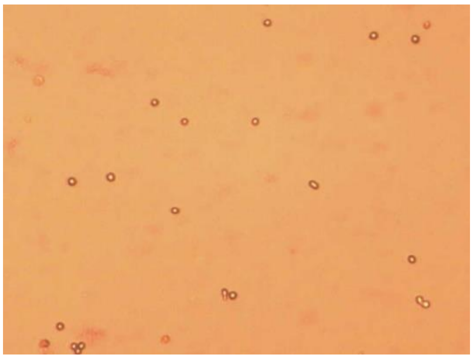

[f] 100 µg/mL

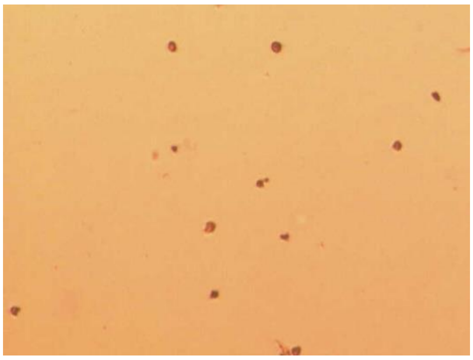

[g] 500 µg/mL

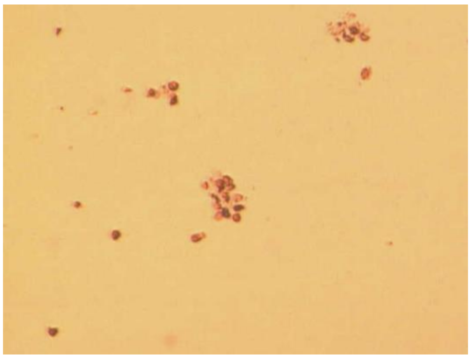

Supplementary Fig. 2/B Effects of PhSeZnCl on cell viability measured by Trypan Blue assay in HepaRG cells after a 24-hour exposure.

[a] Negative control

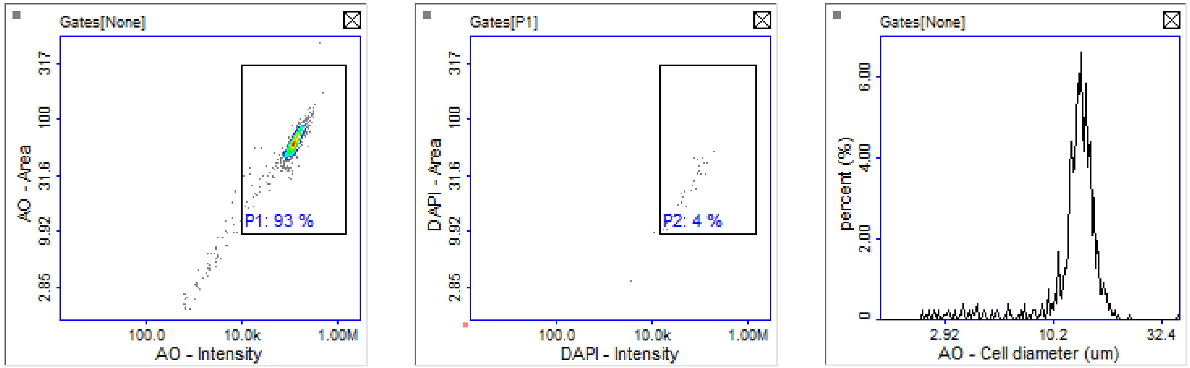

Gate derived results

Viability (%)	95.7
Live (cells/ml)	3.18E5
Dead (cells/ml)	1.44E4
Total (cells/ml)	3.32E5

[b] 6.25 µg/mL

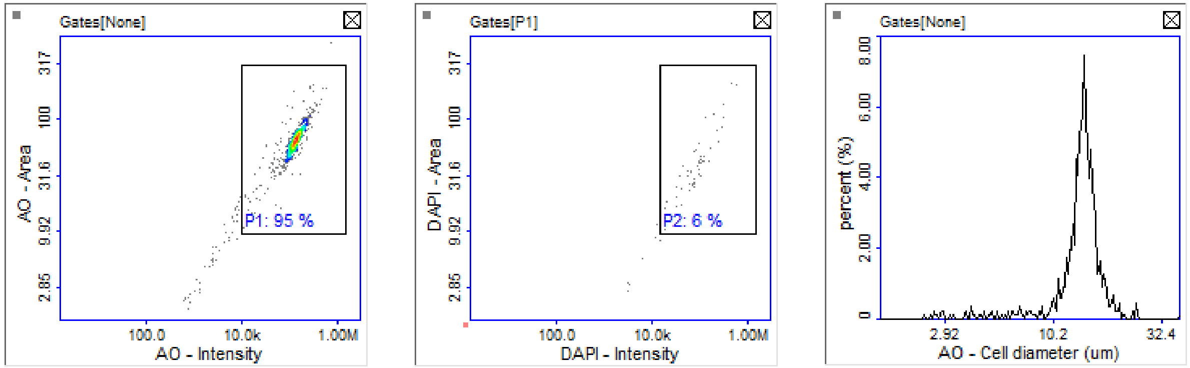

Gate derived results

Viability (%)	93.7
Live (cells/ml)	3.53E5
Dead (cells/ml)	2.36E4
Total (cells/ml)	3.77E5

[c] 12.5 µg/mL

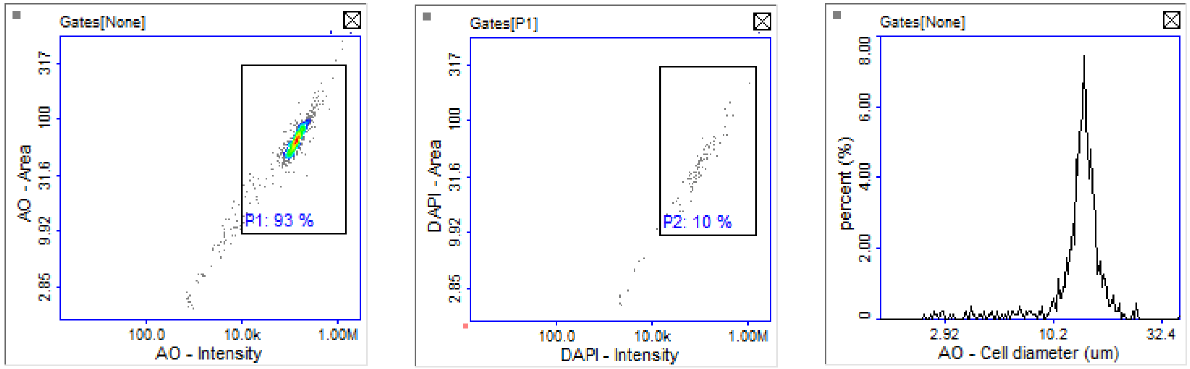

Gate derived results

Viability (%)	90.0
Live (cells/ml)	3.51E5
Dead (cells/ml)	3.89E4
Total (cells/ml)	3.90E5

Supplementary Fig. 3/A (pt. 1) Effects of PhSeZnCl on cell viability measured by AO/DAPI assay in HepG2 cells after a 4-hour exposure.

[d] 25 µg/mL

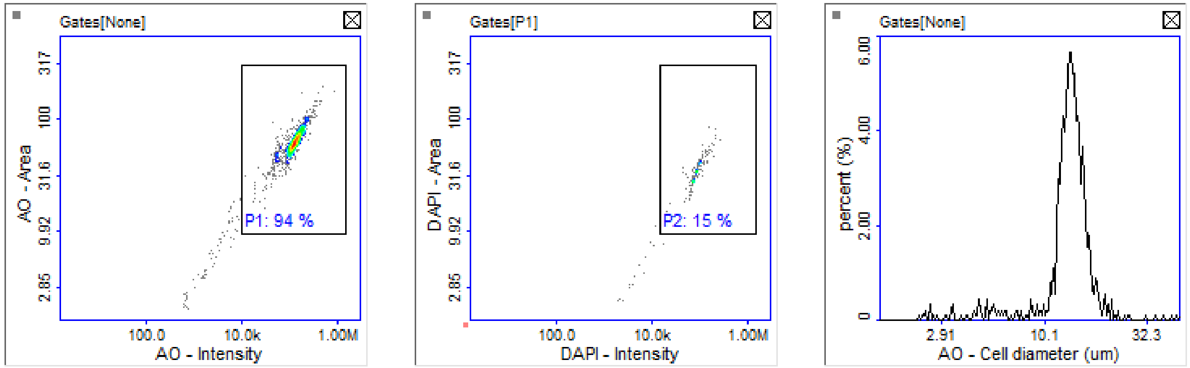

Gate derived results

Viability (%)	84.5
Live (cells/ml)	3.54E5
Dead (cells/ml)	6.48E4
Total (cells/ml)	4.19E5

[e] 50 µg/mL

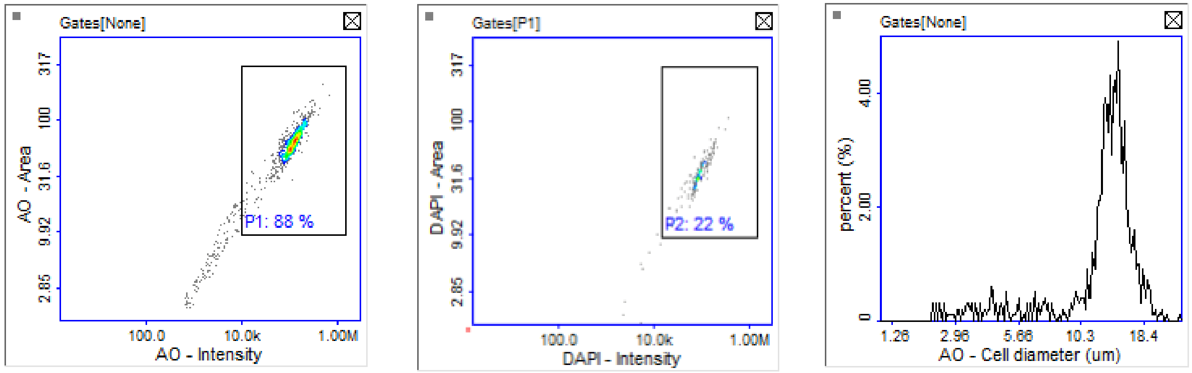

Gate derived results

Viability (%)	77.8
Live (cells/ml)	3.17E5
Dead (cells/ml)	9.03E4
Total (cells/ml)	4.07E5

[f] 100 µg/mL

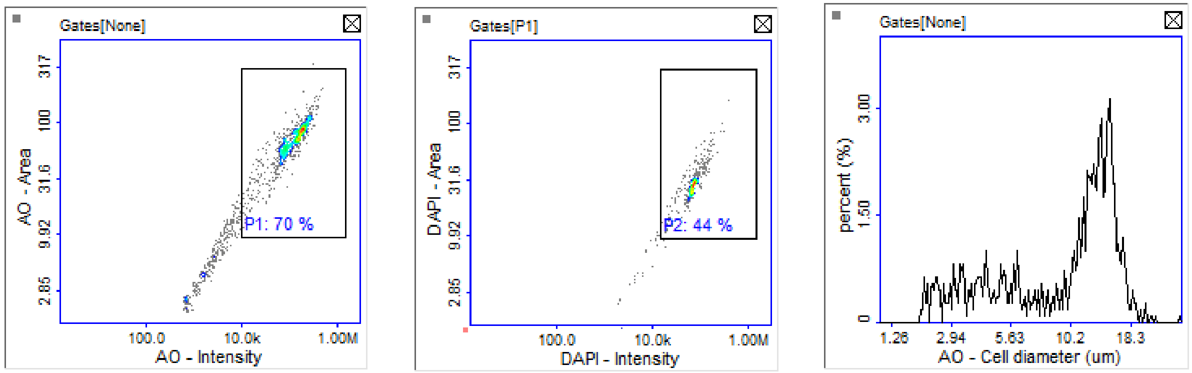

Gate derived results

Viability (%)	56.4
Live (cells/ml)	1.97E5
Dead (cells/ml)	1.52E5
Total (cells/ml)	3.50E5

Supplementary Fig. 3/A (pt. 2) Effects of PhSeZnCl on cell viability measured by AO/DAPI assay in HepG2 cells after a 4-hour exposure.

[g] 500 µg/mL

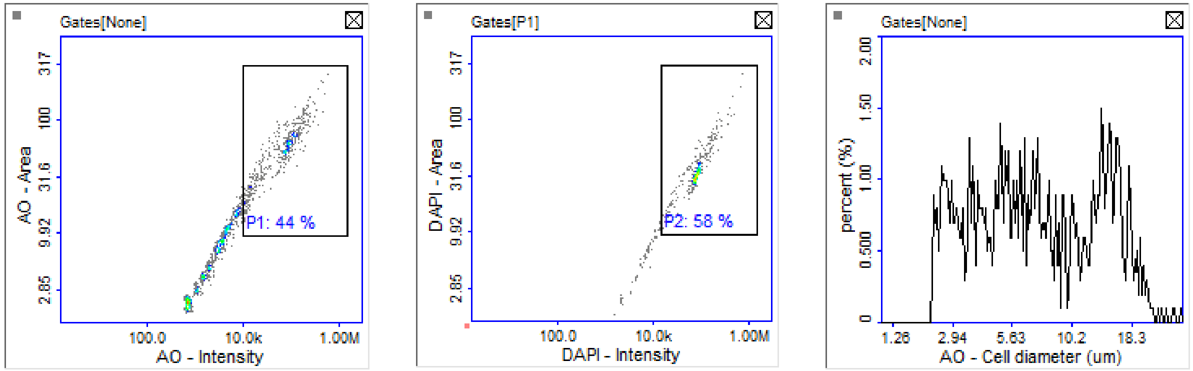

Gate derived results

Viability (%)	42.2
Live (cells/ml)	8.71E4
Dead (cells/ml)	1.19E5
Total (cells/ml)	2.07E5

Supplementary Fig. 3/A (pt. 3) Effects of PhSeZnCl on cell viability measured by AO/DAPI assay in HepG2 cells after a 4-hour exposure.

[a] Negstive control

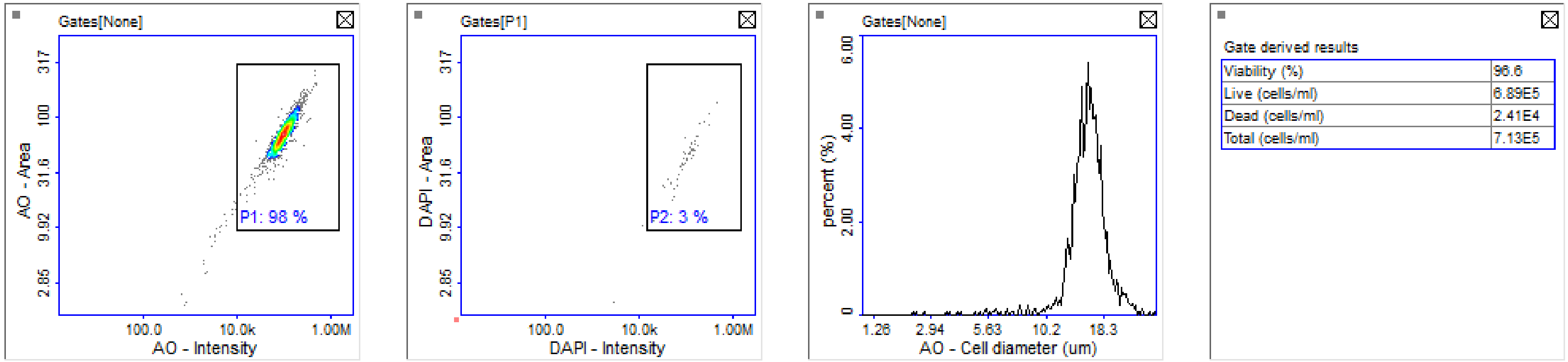

[b] 6.25 µg/mL

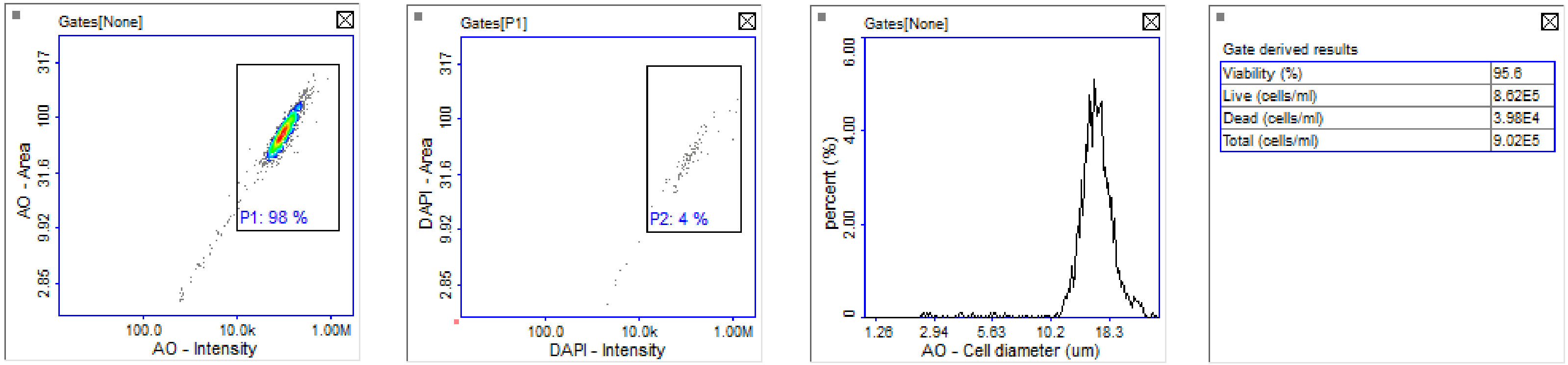

[c] 12.5 µg/mL

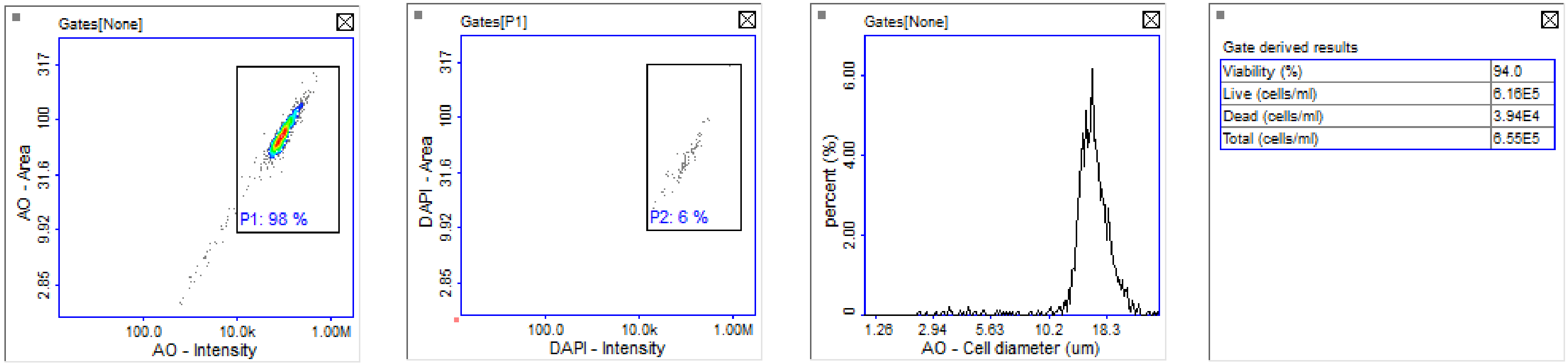

Supplementary Fig. 3/B (pt. 1) Effects of PhSeZnCl on cell viability measured by AO/DAPI assay in HepG2 cells after a 24-hour exposure.

[d] 25 µg/mL

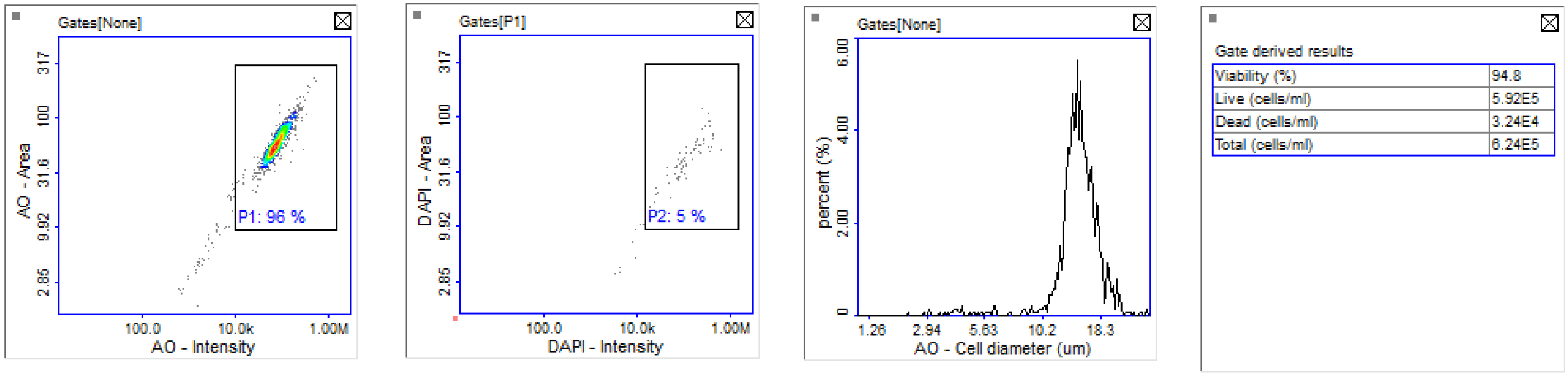

[e] 50 µg/mL

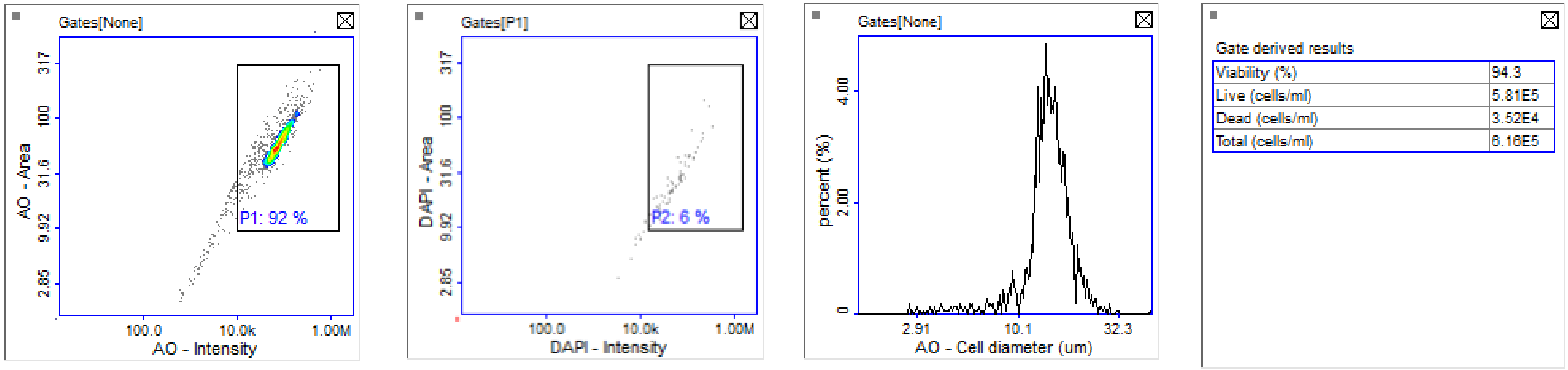

[f] 100 µg/mL

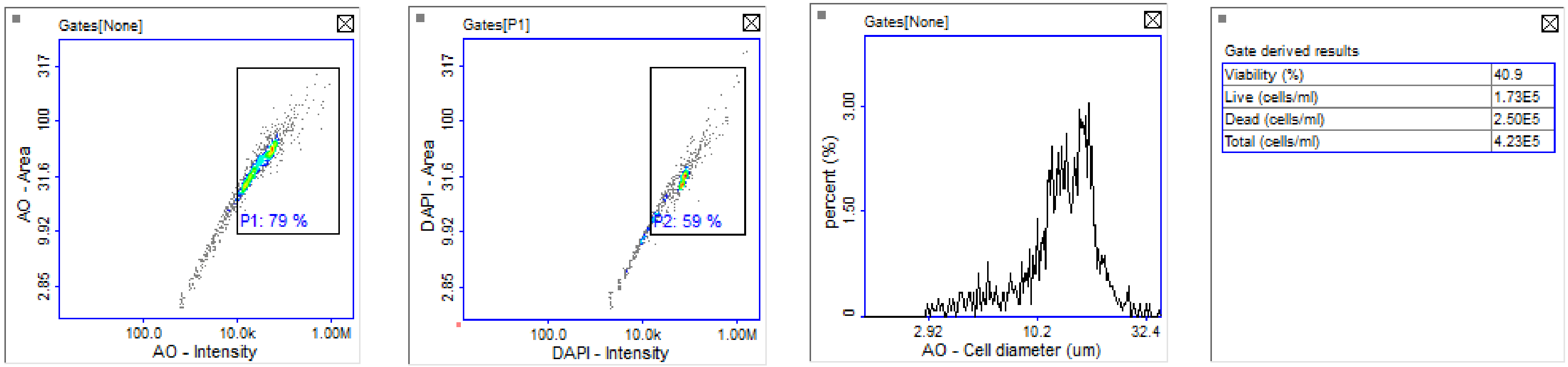

Supplementary Fig. 3/B (pt. 2) Effects of PhSeZnCl on cell viability measured by AO/DAPI assay in HepG2 cells after a 24-hour exposure.

[g] 500 μg/mL

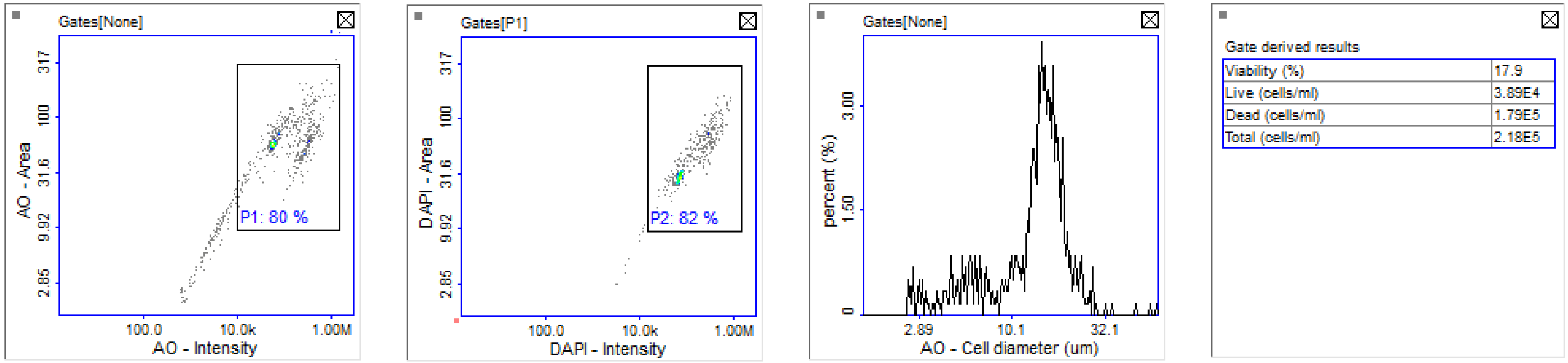

Supplementary Fig. 3/B (pt. 3) Effects of PhSeZnCl on cell viability measured by AO/DAPI assay in HepG2 cells after a 24-hour exposure.

[a] Negative control

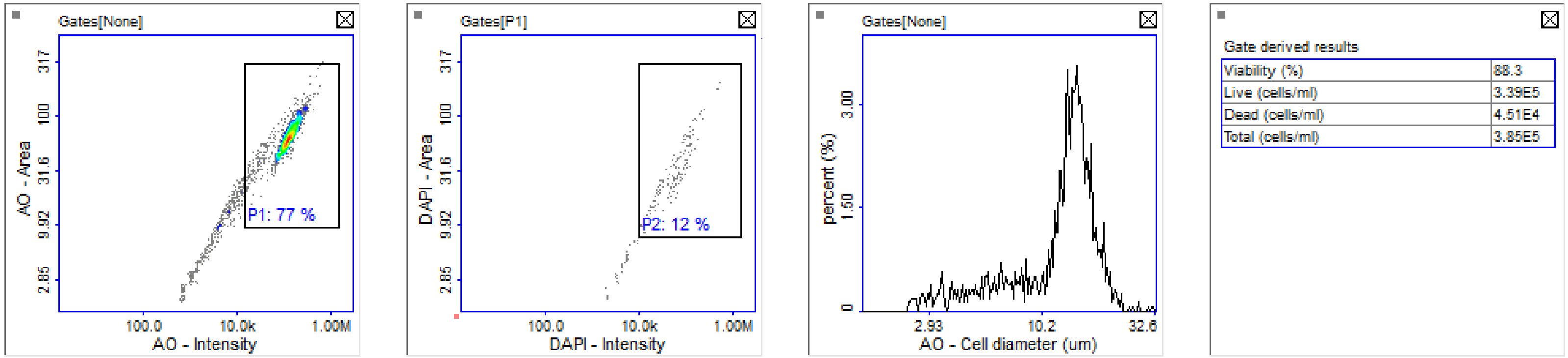

[b] 6.25 µg/mL

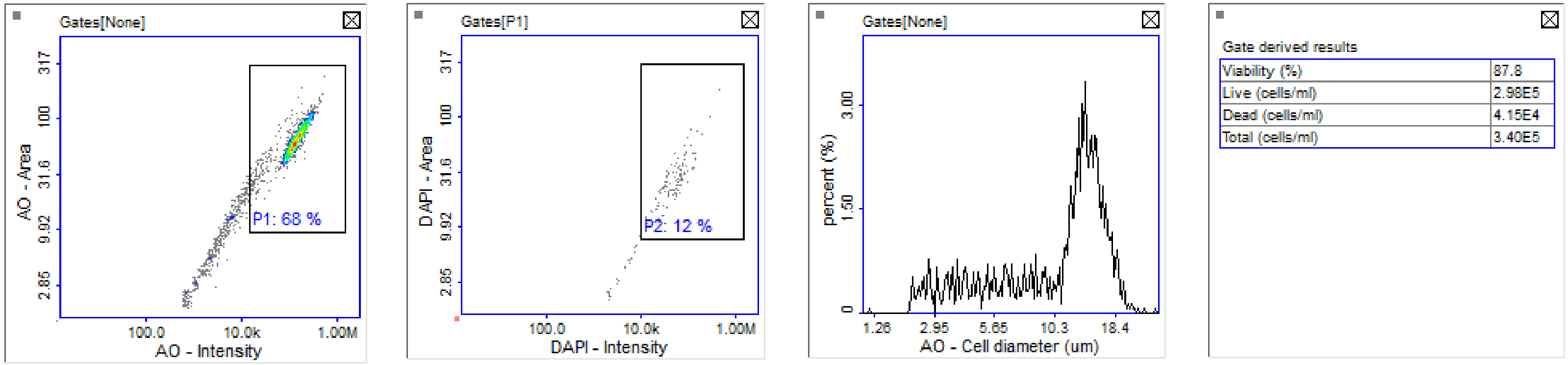

[c] 12.5 µg/mL

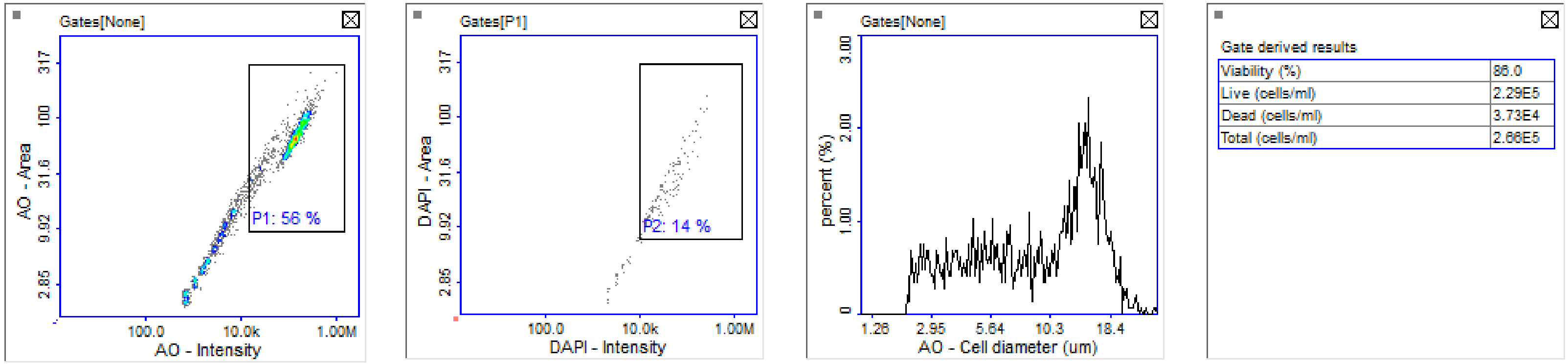

Supplementary Fig. 4/A (pt. 1) Effects of PhSeZnCl on cell viability measured by AO/DAPI assay in HepaRG cells after a 4-hour exposure.

[d] 25 µg/mL

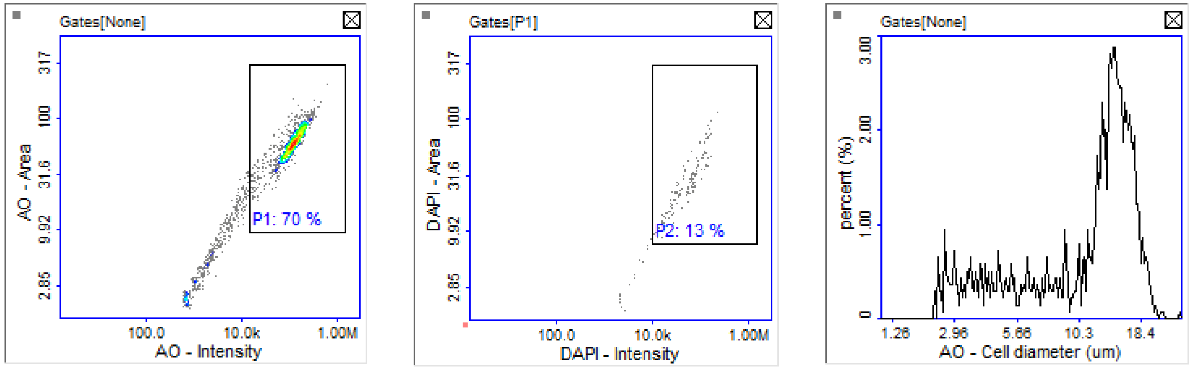

Gate derived results

Viability (%)	86.9
Live (cells/ml)	2.76E5
Dead (cells/ml)	4.15E4
Total (cells/ml)	3.18E5

[e] 50 µg/mL

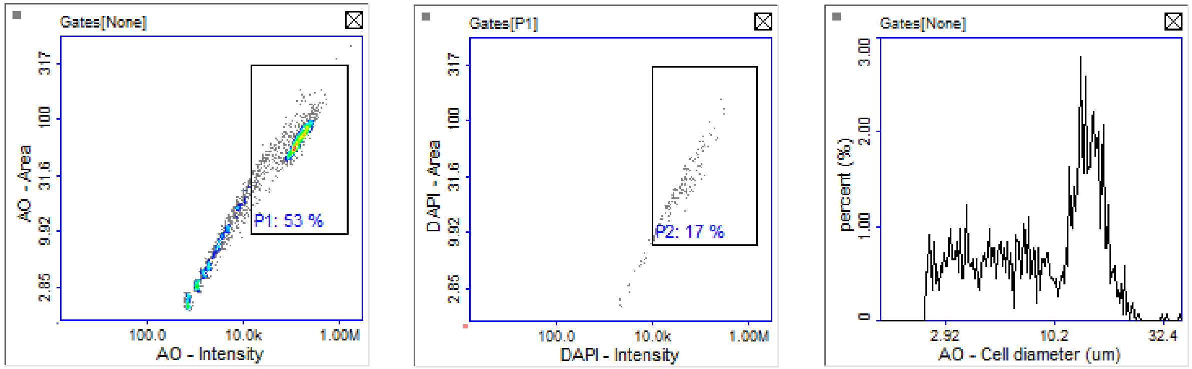

Gate derived results

Viability (%)	82.9
Live (cells/ml)	2.18E5
Dead (cells/ml)	4.51E4
Total (cells/ml)	2.63E5

[f] 100 µg/mL

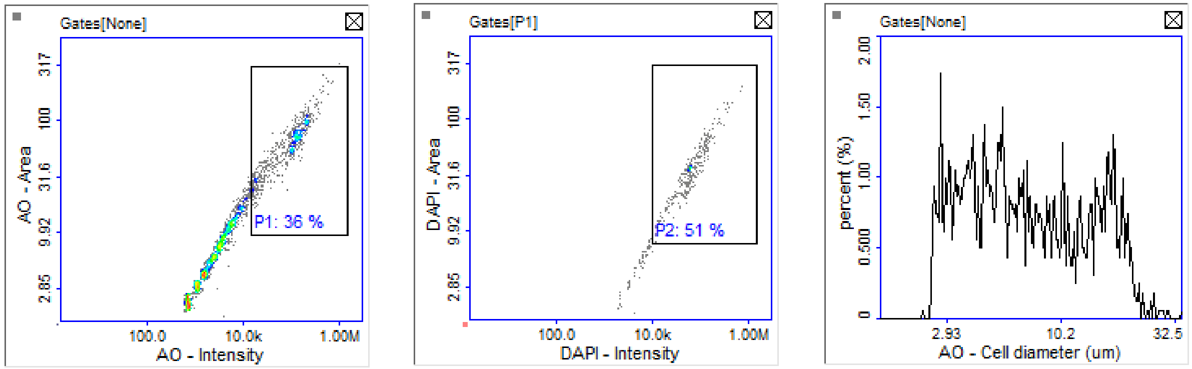

Gate derived results

Viability (%)	48.8
Live (cells/ml)	9.18E4
Dead (cells/ml)	9.63E4
Total (cells/ml)	1.88E5

Supplementary Fig. 4/A (pt. 2) Effects of PhSeZnCl on cell viability measured by AO/DAPI assay in HepaRG cells after a 4-hour exposure.

[g] 500 µg/mL

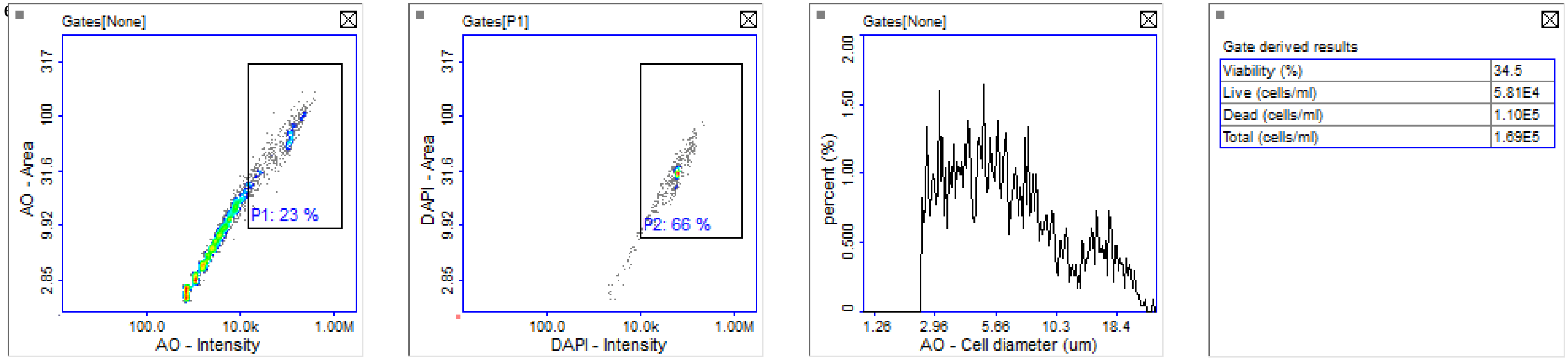

Supplementary Fig. 4/A (pt. 3) Effects of PhSeZnCl on cell viability measured by AO/DAPI assay in HepaRG cells after a 4-hour exposure.

[a] Negatoive control

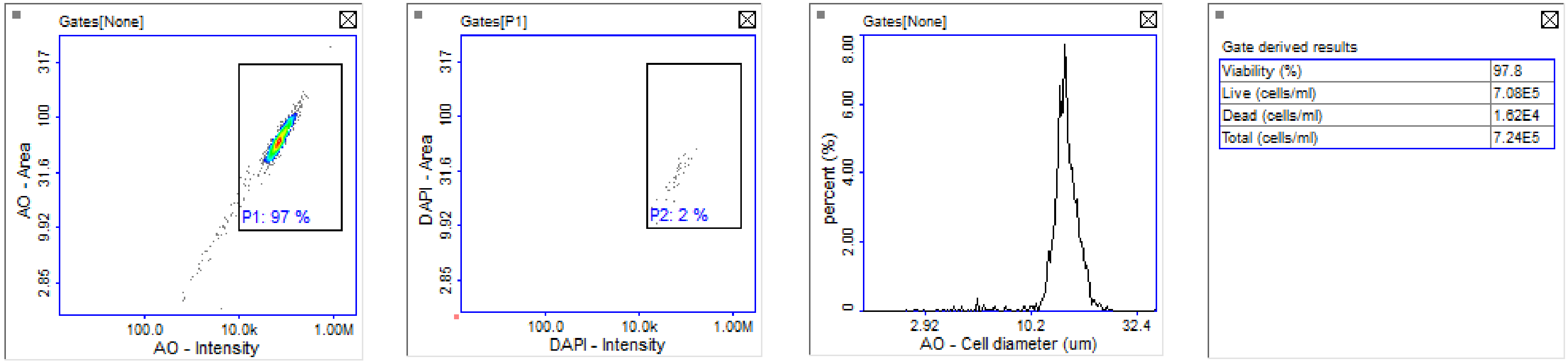

[b] 6.25 µg/mL

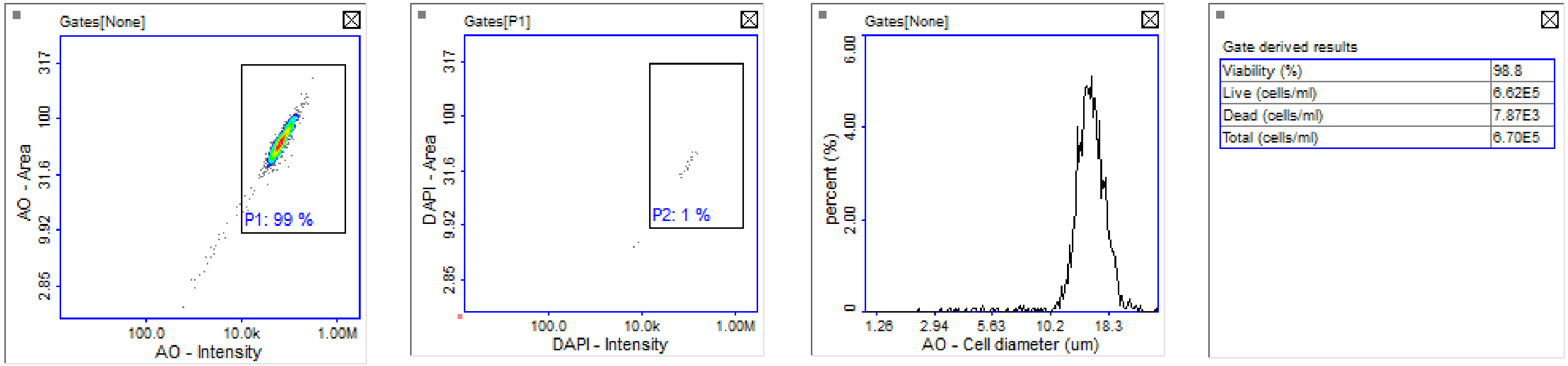

[c] 12.5 µg/mL

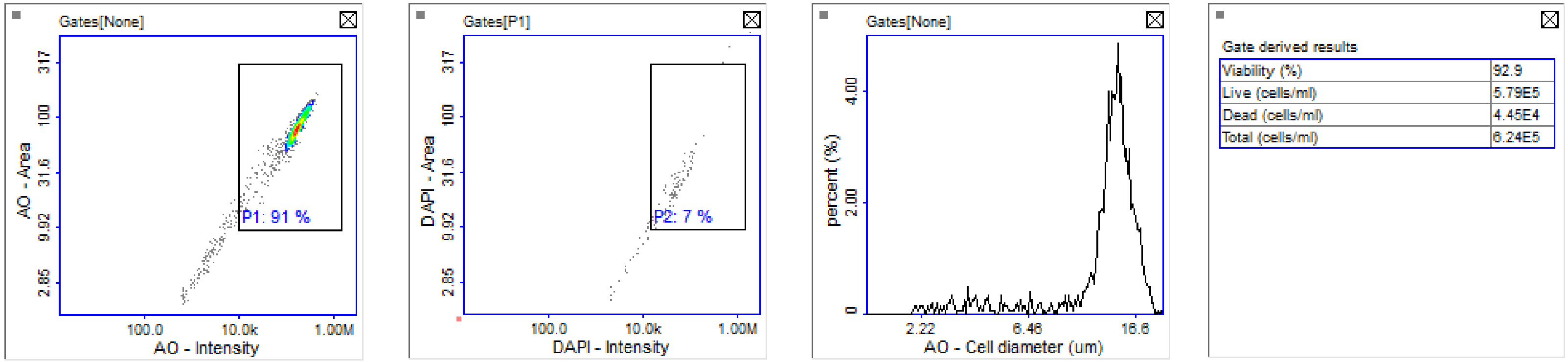

Supplementary Fig. 4/B (pt. 1) Effects of PhSeZnCl on cell viability measured by AO/DAPI assay in HepaRG cells after a 24-hour exposure.

[d] 25 µg/mL

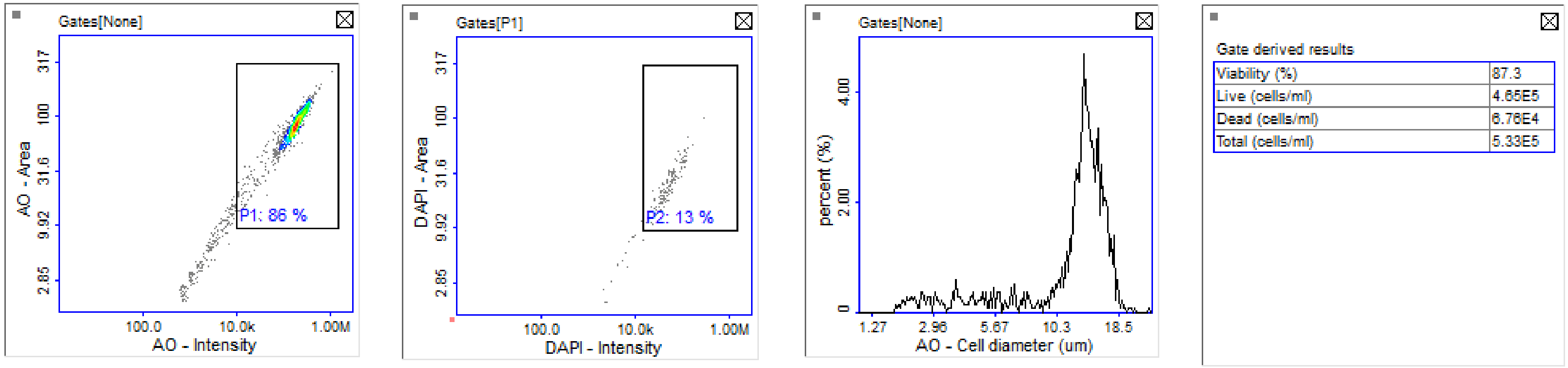

[e] 50 µg/mL

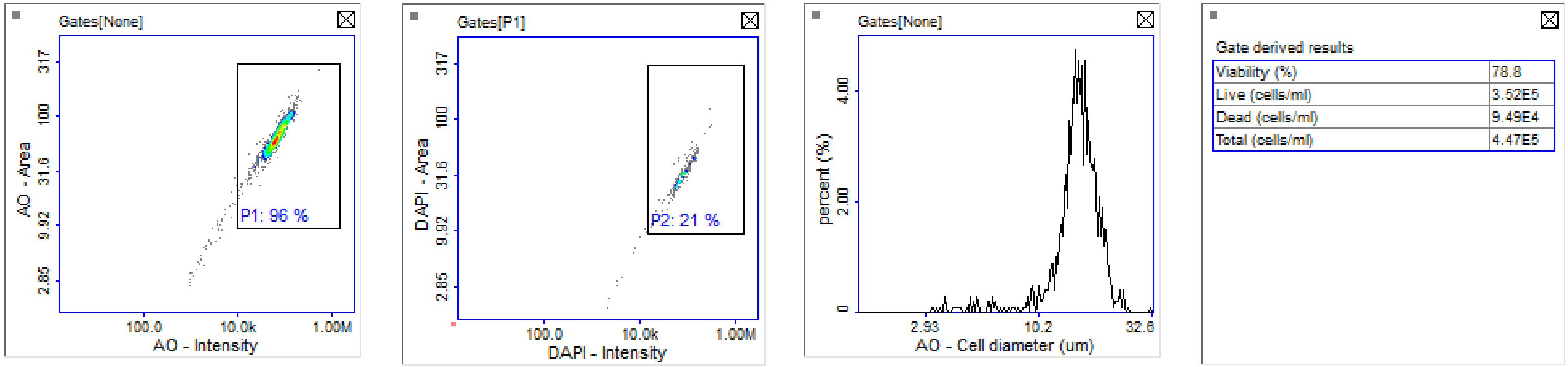

[f] 100 µg/mL

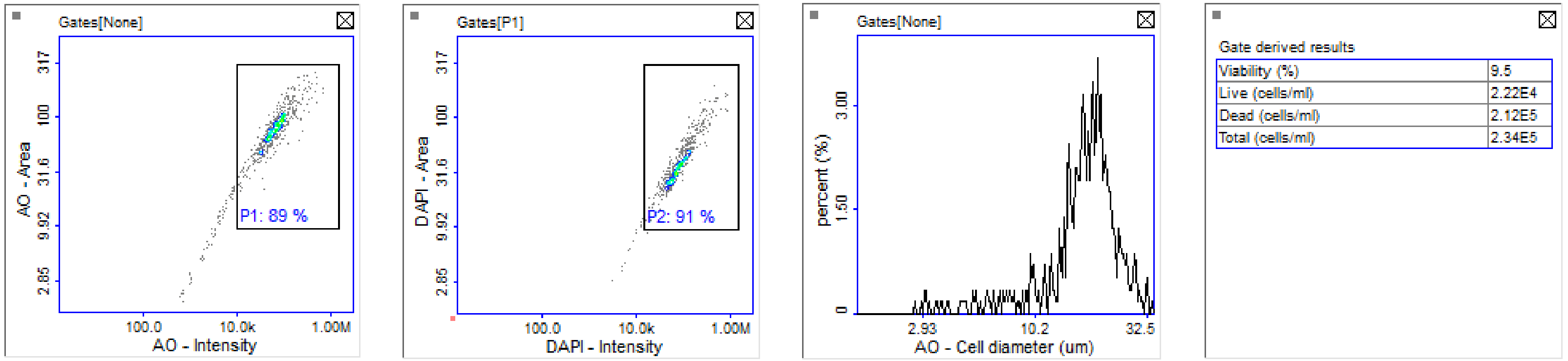

Supplementary Fig. 4/B (pt. 2) Effects of PhSeZnCl on cell viability measured by AO/DAPI assay in HepaRG cells after a 24-hour exposure.

[g] 500 µg/mL

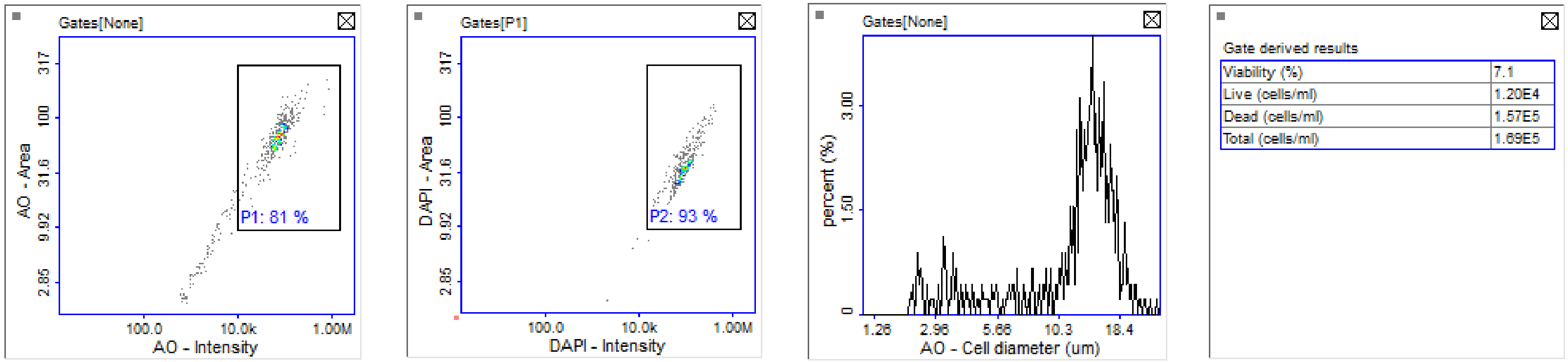

Supplementary Fig. 4/B (pt. 3) Effects of PhSeZnCl on cell viability measured by AO/DAPI assay in HepaRG cells after a 24-hour exposure.

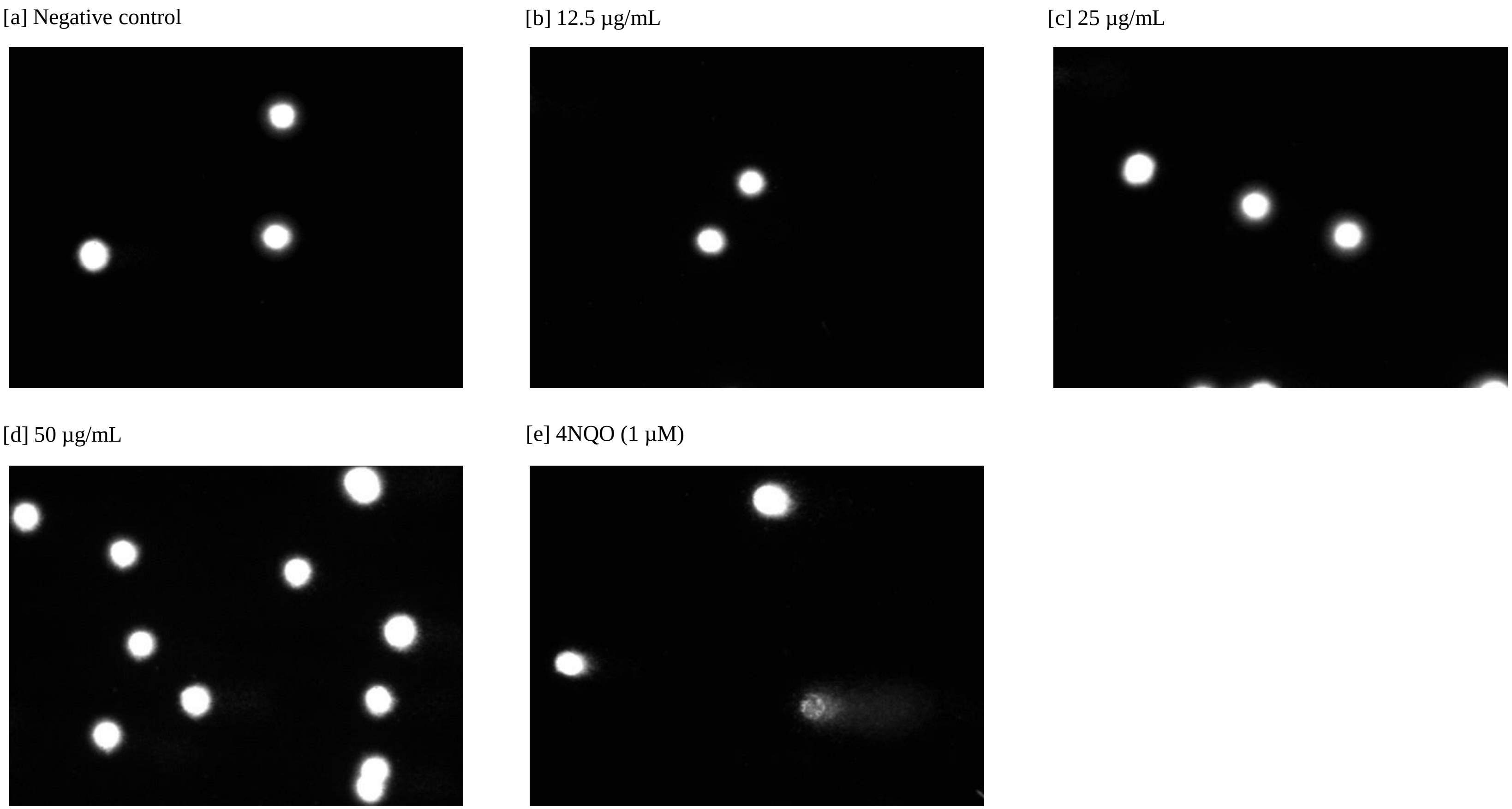

Supplementary Fig. 5 Primary DNA damage in HepG2 cells exposed for 4 hours to the three highest concentrations of PhSeZnCl which did not show cytotoxic effects.

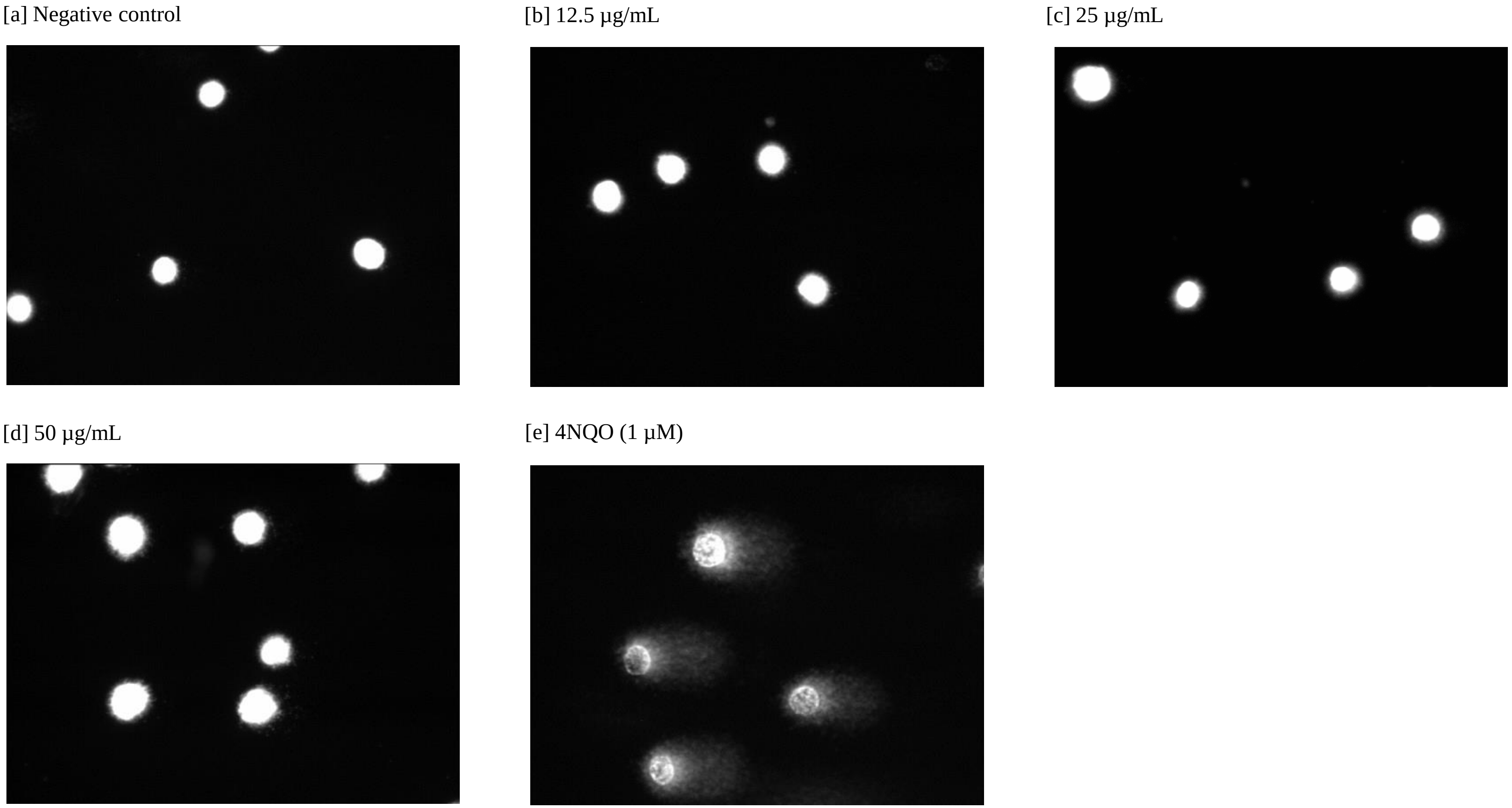

Supplementary Fig. 6 Primary DNA damage in HepaRG cells exposed for 4 hours to the three highest concentrations of PhSeZnCl which did not show cytotoxic effects.

[a] Negative control

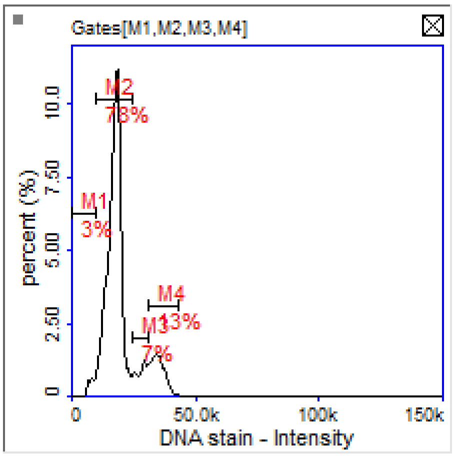

[b] 12.5 µg/mL

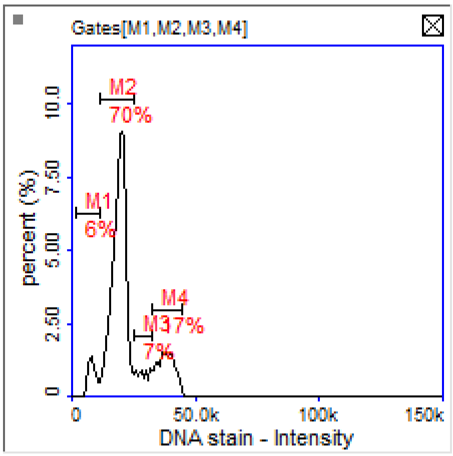

[c] 25 µg/mL

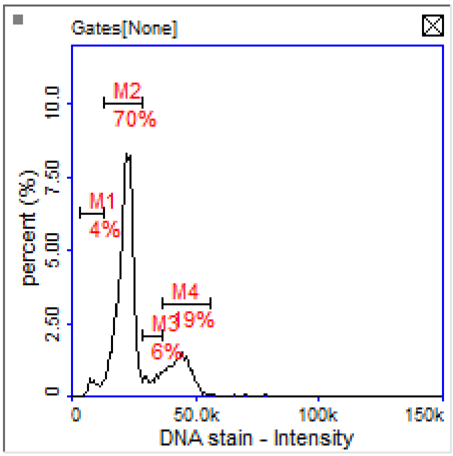

[d] 50 µg/mL

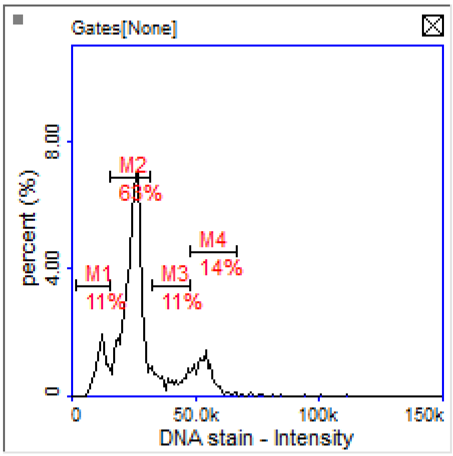

Supplementary Fig. 7 Effects of PhSeZnCl on HepG2 cell cycle after a 24-hour exposure determined using the three highest concentrations which did not show cytotoxic effects.

[a] Negative control

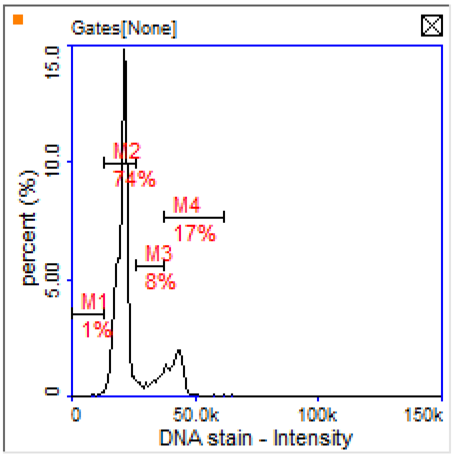

[b] 12.5 µg/mL

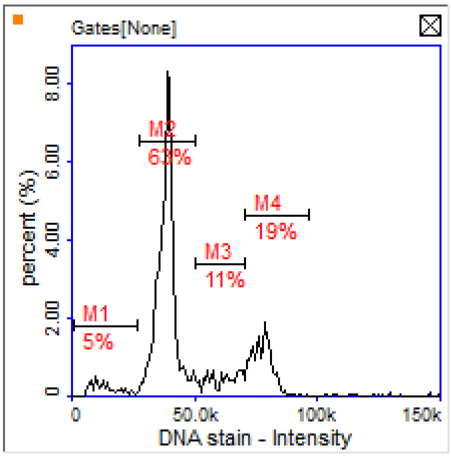

[c] 25 µg/mL

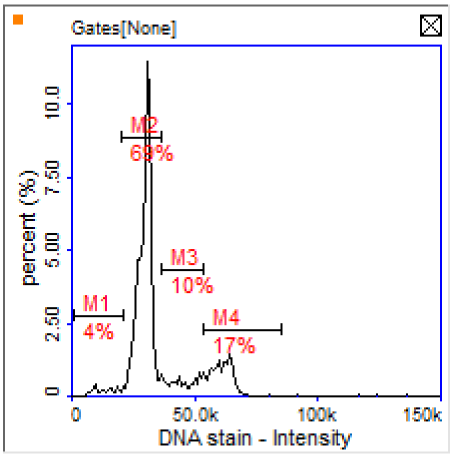

[d] 50 µg/mL

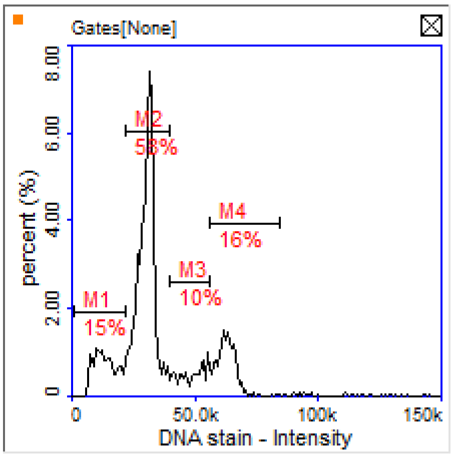

Supplementary Fig. 8 Effects of PhSeZnCl on HepaRG cell cycle after a 24-hour exposure determined using the three highest concentrations which did not show cytotoxic effects.

[a] Negative control

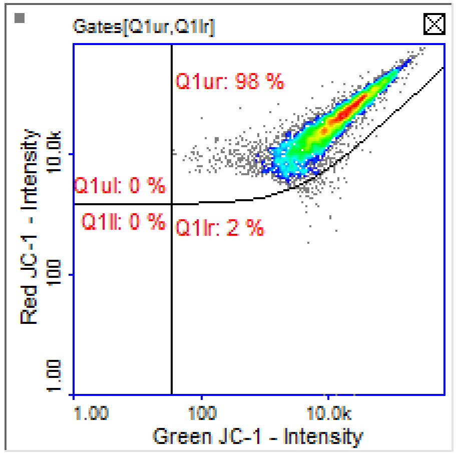

[b] 12.5 µg/mL

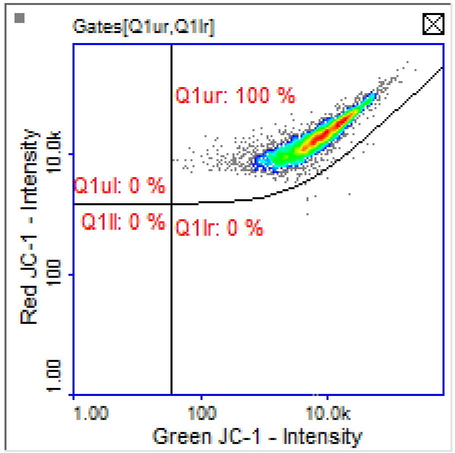

[c] 25 µg/mL

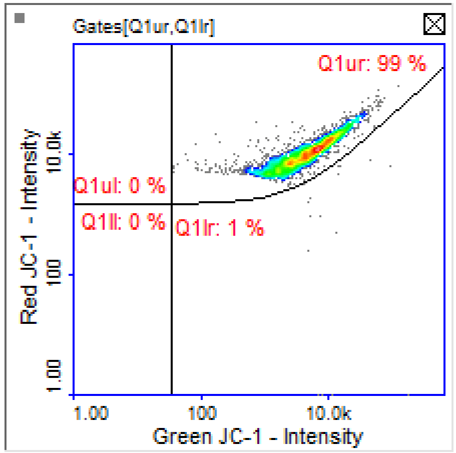

[d] 50 µg/mL

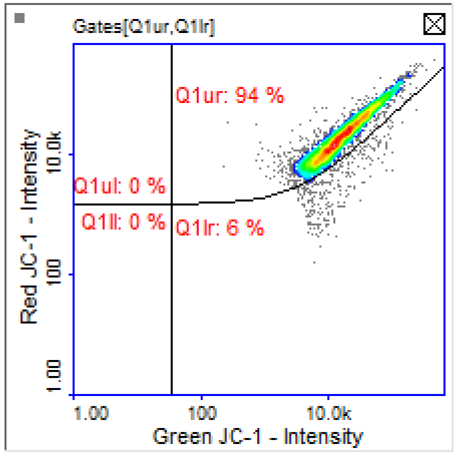

[e] Valinomycin (0.5 µM)

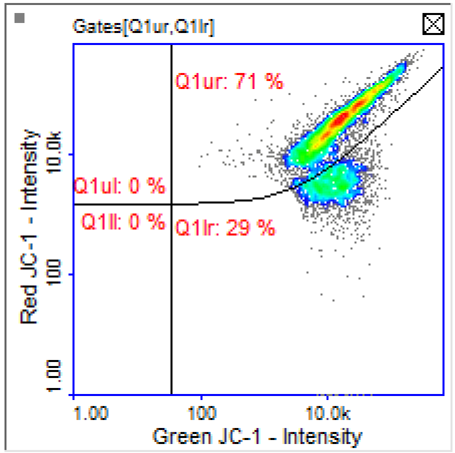

Supplementary Fig. 9/A Effects of PhSeZnCl on early (mitochondrial membrane depolarization; ΔΨm) apoptosis induction in HepG2 cells after a 4-hour exposure.

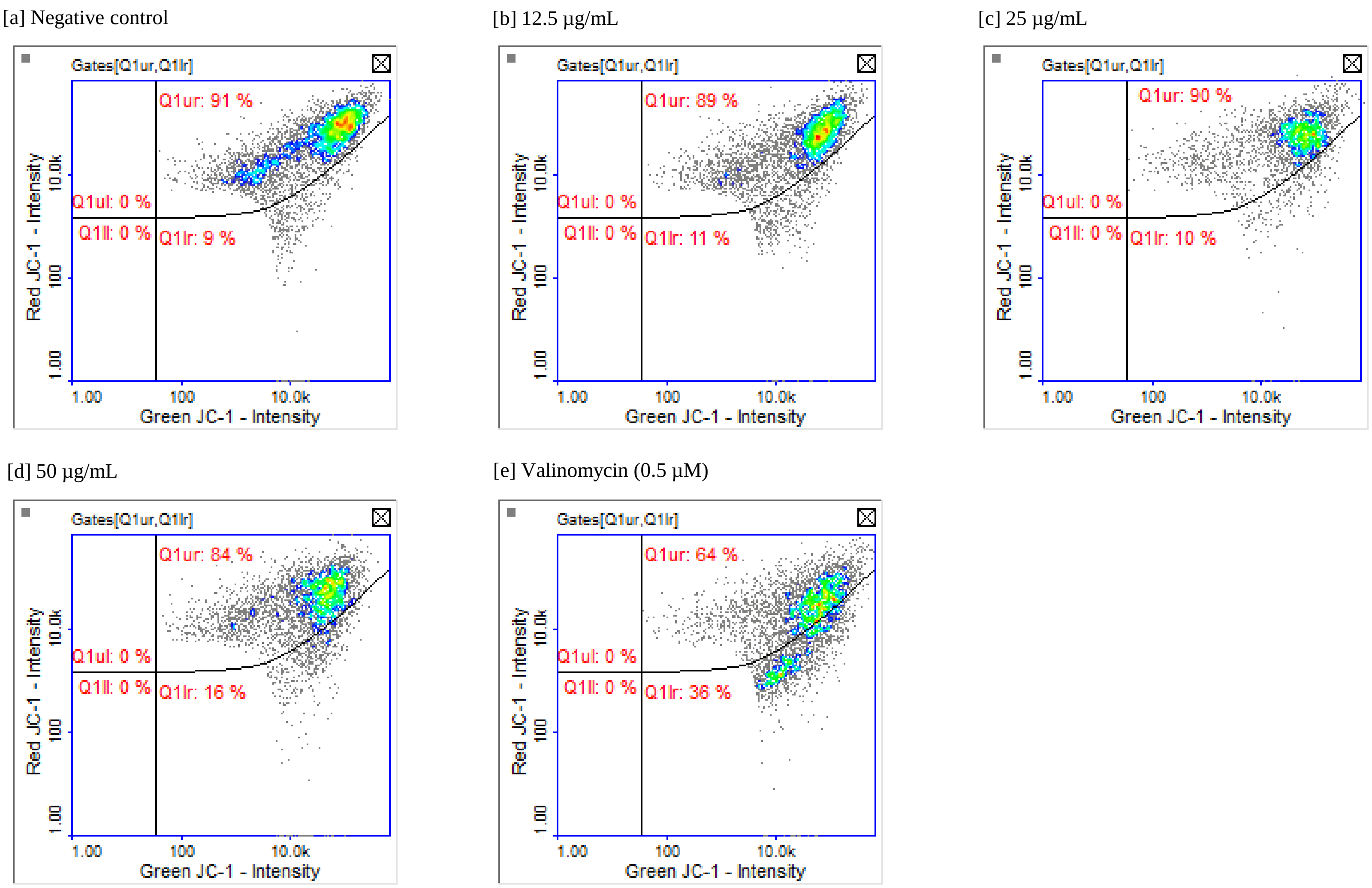

Supplementary Fig. 9/B Effects of PhSeZnCl on early (mitochondrial membrane depolarization; ΔΨm) apoptosis induction in HepG2 cells after a 24-hour exposure.

[a] Negative control

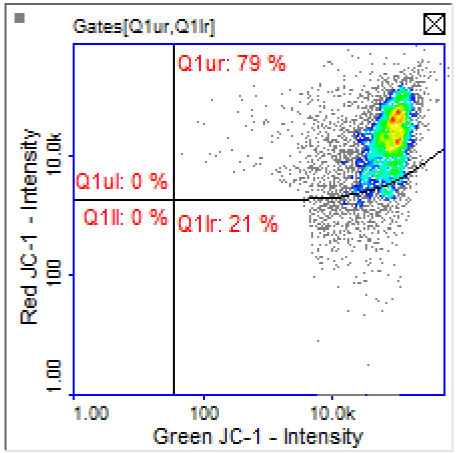

[b] 12.5 µg/mL

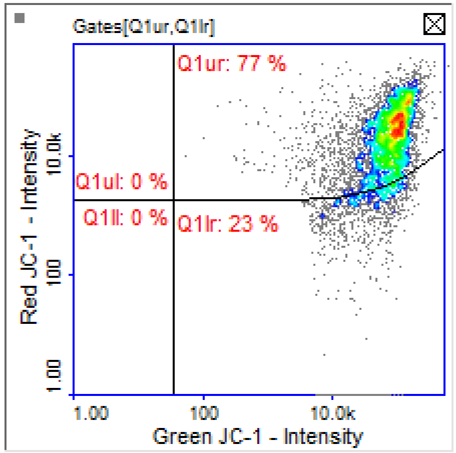

[c] 25 µg/mL

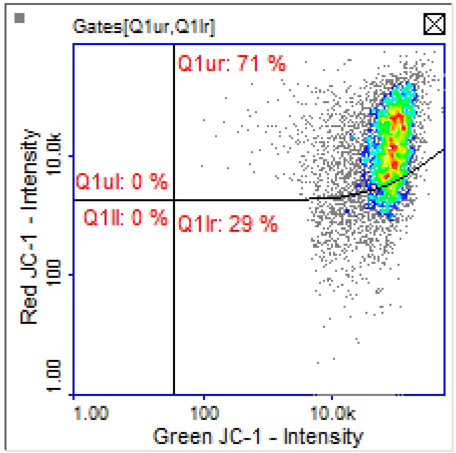

[d] 50 µg/mL

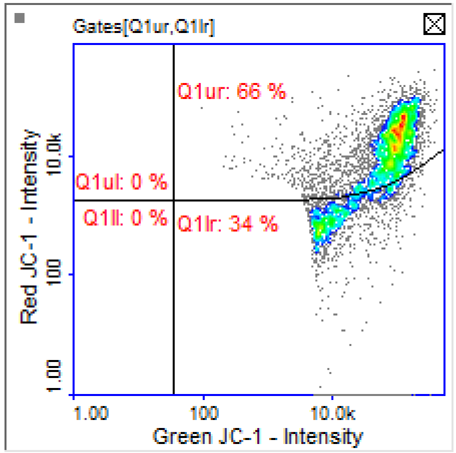

[e] Valinomycin (0.5 µM)

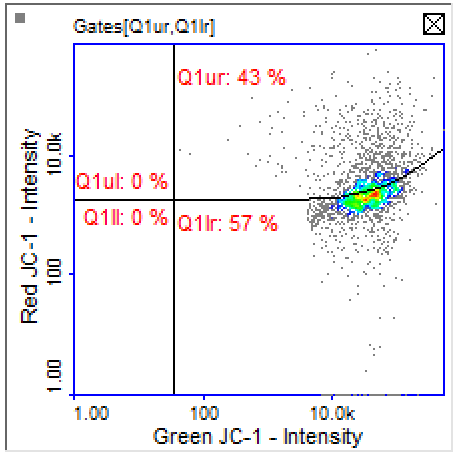

Supplementary Fig. 10/A Effects of PhSeZnCl on early (mitochondrial membrane depolarization; ΔΨm) apoptosis induction in HepaRG cells after a 4-hour exposure.

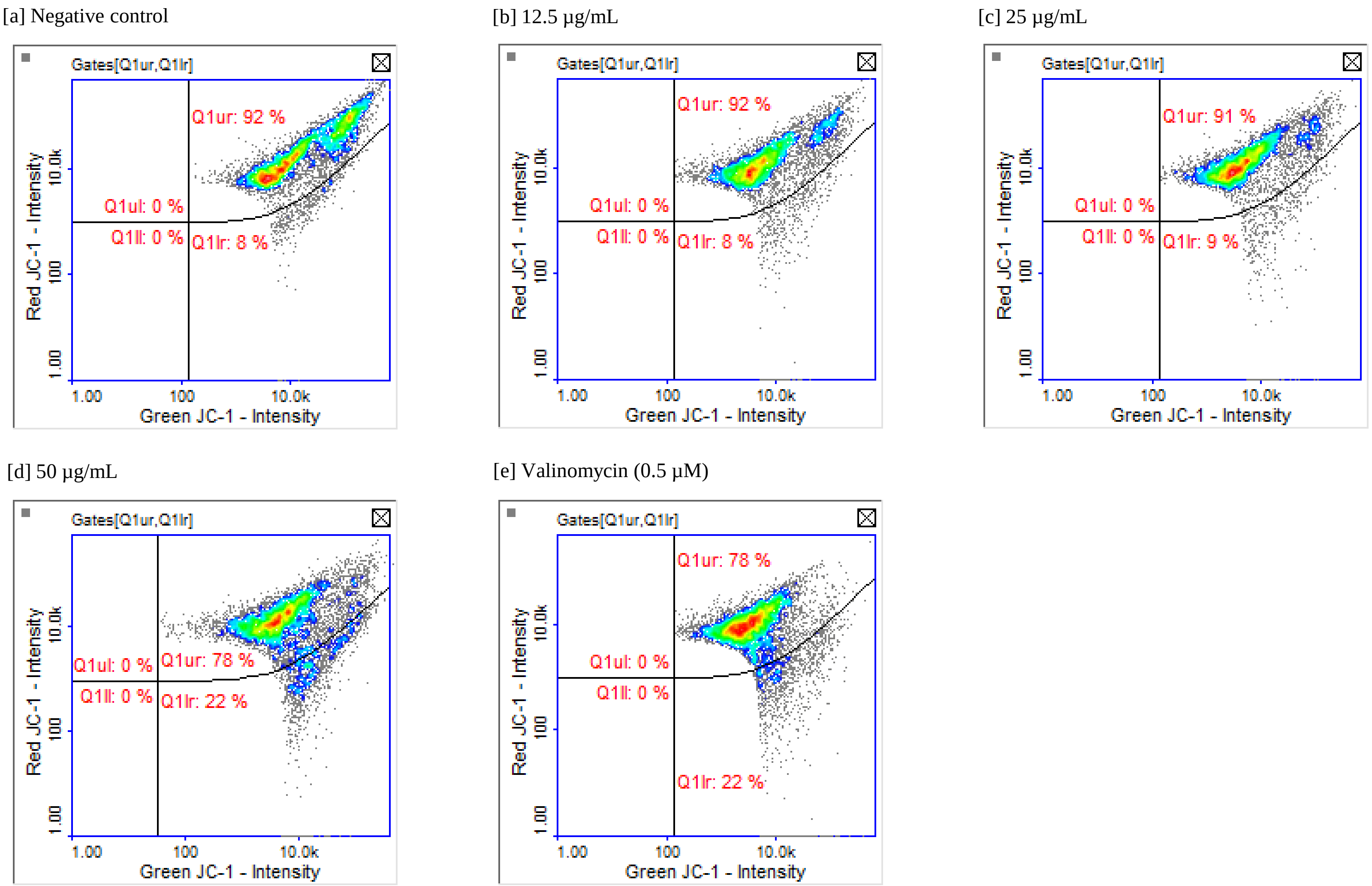

Supplementary Fig. 10/B Effects of PhSeZnCl on early (mitochondrial membrane depolarization; ΔΨm) apoptosis induction in HepaRG cells after a 24-hour exposure.

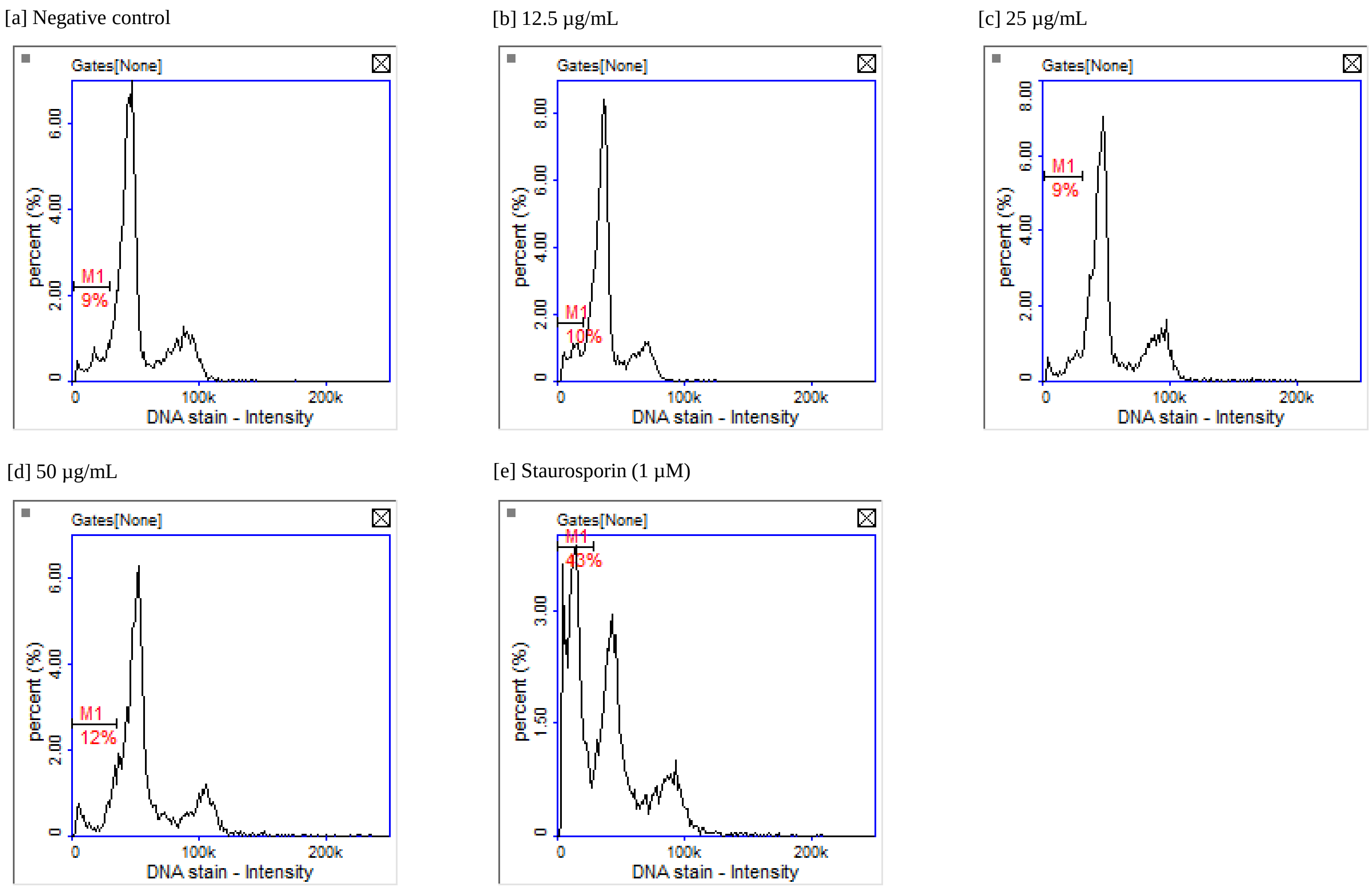

Supplementary Fig. 11/A Effects of PhSeZnCl on late (chromosomal DNA fragmentation) apoptosis induction in HepG2 cells after a 4-hour exposure.

[a] Negative control

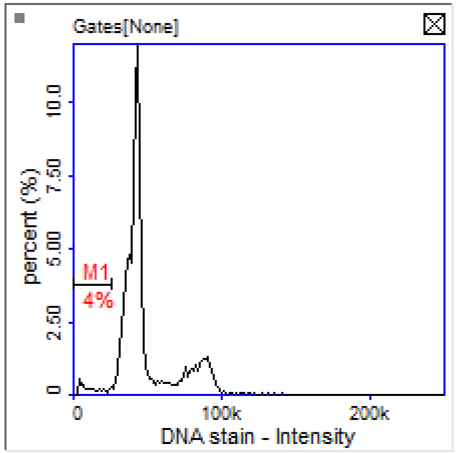

[b] 12.5 µg/mL

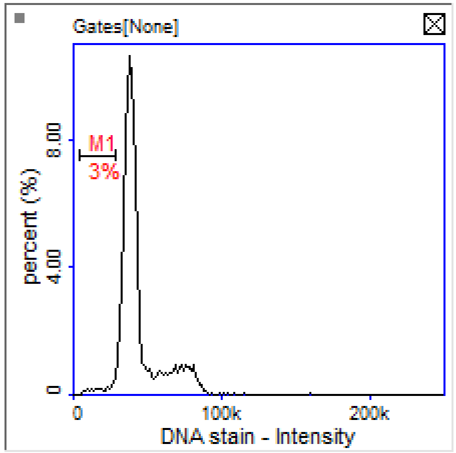

[c] 25 µg/mL

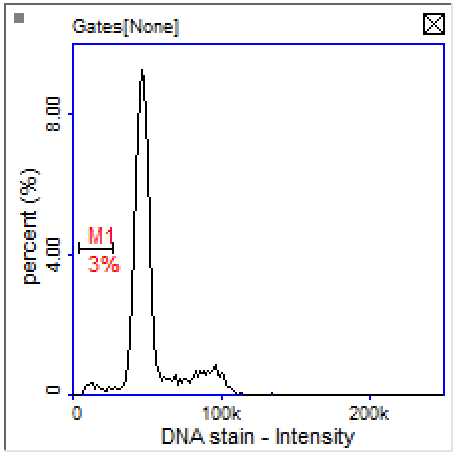

[d] 50 µg/mL

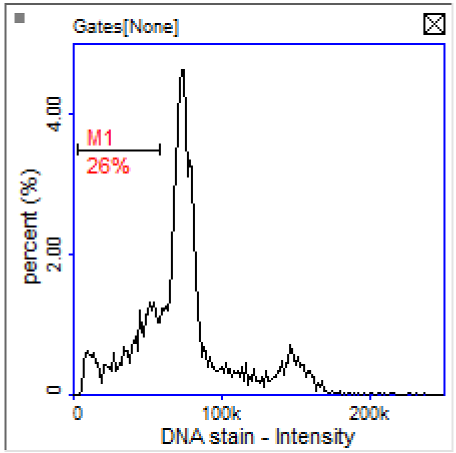

[e] Staurosporin (1 µM)

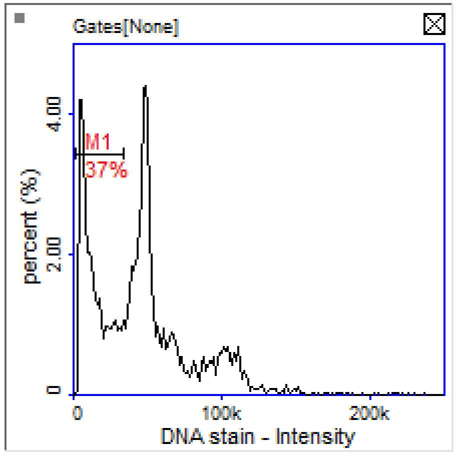

Supplementary Fig. 11/B Effects of PhSeZnCl on late (chromosomal DNA fragmentation) apoptosis induction in HepG2 cells after a 24-hour exposure.

[a] Negative control

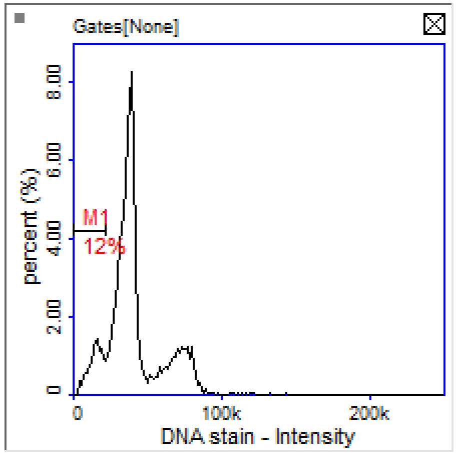

[b] 12.5 µg/mL

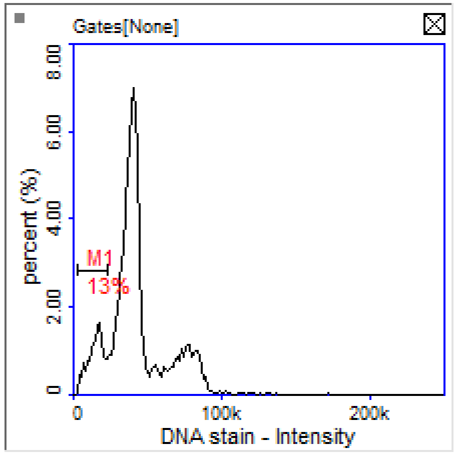

[c] 25 µg/mL

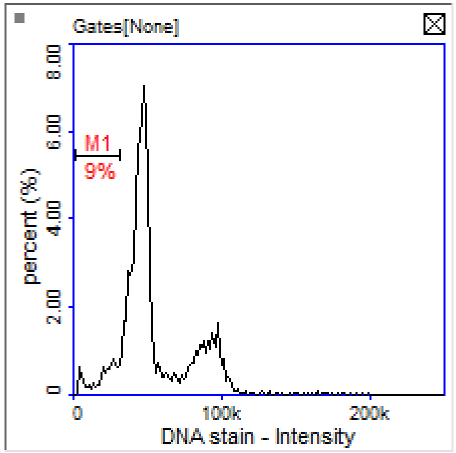

[d] 50 µg/mL

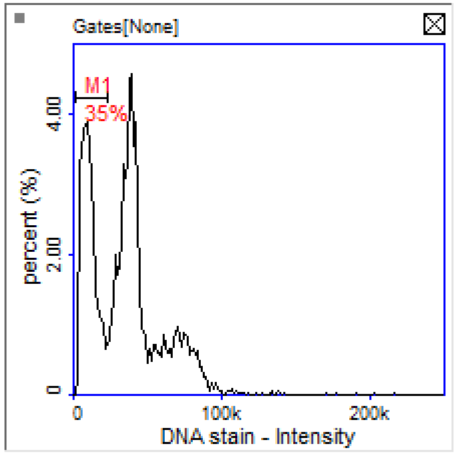

[e] Staurosporin (1 µM)

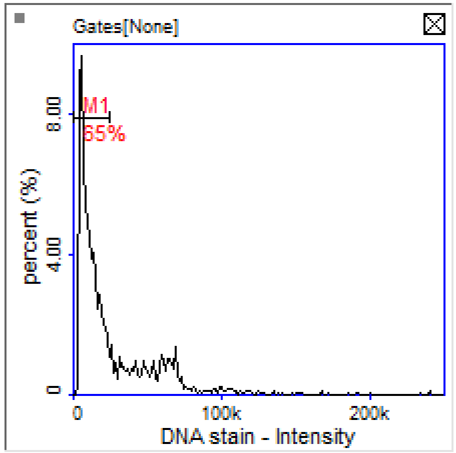

Supplementary Fig. 12/A Effects of PhSeZnCl on late (chromosomal DNA fragmentation) apoptosis induction in HepaRG cells after a 4-hour exposure.

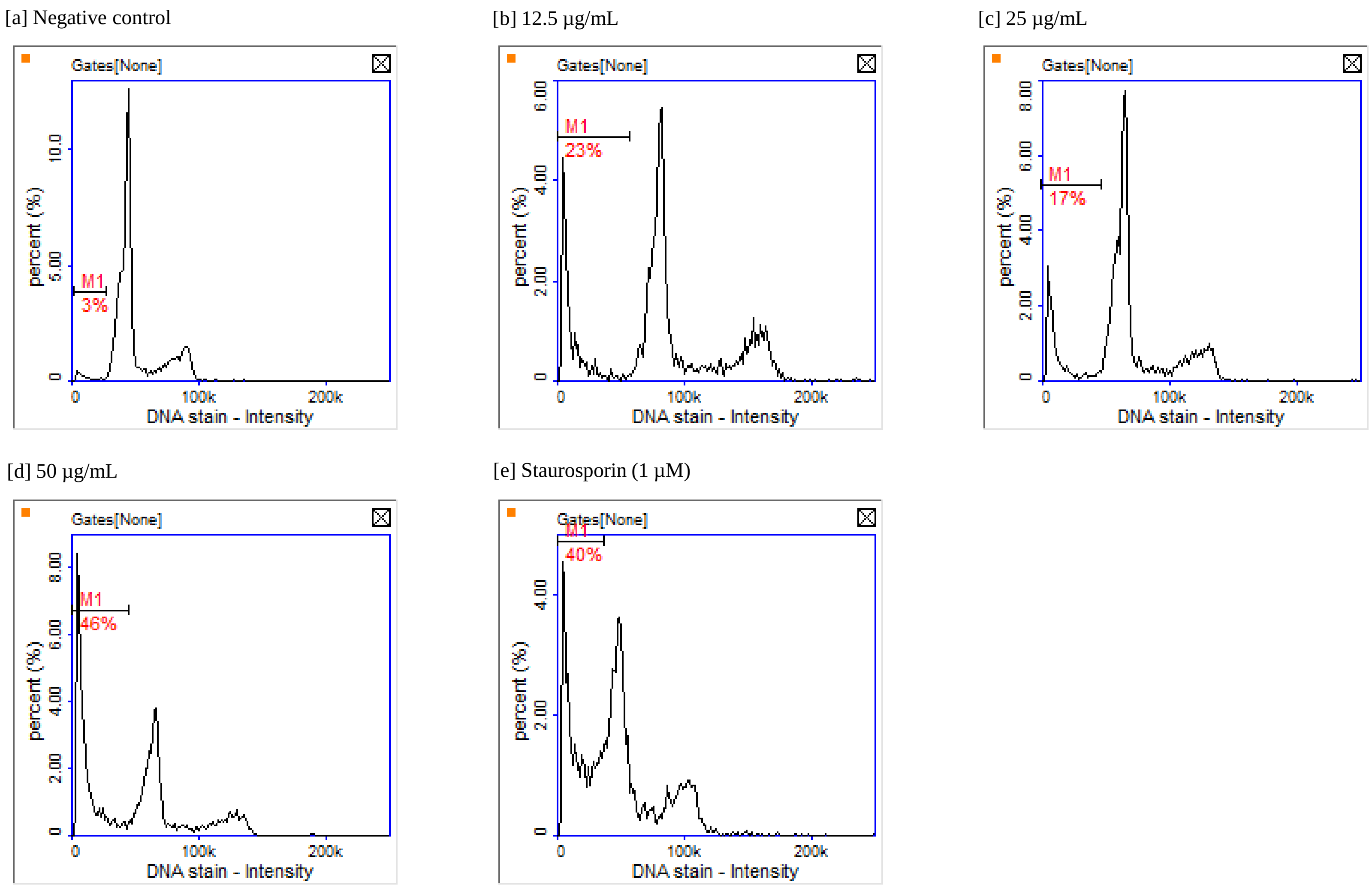

Supplementary Fig. 12/B Effects of PhSeZnCl on late (chromosomal DNA fragmentation) apoptosis induction in HepaRG cells after a 24-hour exposure.
